# Associations of cerebrospinal fluid complement proteins with Alzheimer’s pathology, cognition, and brain structure in non-dementia elderly

**DOI:** 10.1186/s13195-023-01377-5

**Published:** 2024-01-18

**Authors:** Meng Li, Ya-Hui Ma, Yun Guo, Jia-Yao Liu, Lan Tan, on behalf of Alzheimer’s Disease Neuroimaging Initiative

**Affiliations:** 1 Department of Neurology, Qingdao Municipal Hospital, Qingdao University, Qingdao, China; 2 Department of Neurology, The Affiliated Hospital of Qingdao University, Qingdao, 266071 China; 3 School of Clinical Medicine, Weifang Medical University, Weifang, China

**Keywords:** AD, Complement, Biomarkers, Cognition, Brain structure

## Abstract

**Background:**

Cerebrospinal fluid (CSF) complement activation is a key part of neuroinflammation that occurs in the early stages of Alzheimer’s disease (AD). However, the associations of CSF complement proteins with AD pathology, cognition, and structural neuroimaging biomarkers for AD have been rarely investigated.

**Methods:**

A total of 210 participants (125 mild cognitive impairment [MCI] patients and 85 normal controls) were included from Alzheimer’s Disease Neuroimaging Initiative (ADNI) database who measured AD pathology, cognition, and neuroimaging at baseline and every 12 months. The mixed-effect linear models were utilized to investigate longitudinal associations of CSF complement proteins with AD pathology, cognition, and neuroimaging in cognitively normal (CN) and mild cognitive impairment (MCI) subjects. Causal mediation analyses were conducted to explore the potential mediators between CSF complement proteins and cognitive changes.

**Results:**

We found that the subjects with low CSF complement protein levels at baseline had worse outcomes in AD pathology, indicated by their lowest concentrations observed in A + and A + T + individuals. The reduced CSF complement proteins were associated with faster accumulation of tau among CN subjects and with cognitive decline and greater brain atrophy of specific regions among MCI subjects. Furthermore, mediation analyses showed that the effects of CSF complement proteins on cognitive performance were partially mediated by regional brain structures (mediation proportions range from 19.78 to 94.92%; *p* < 0.05).

**Conclusions:**

This study demonstrated that CSF complement proteins were involved in the early progression of AD. Our results indicated that regional brain atrophy might be a plausible way to connect CSF complement protein levels and cognition.

**Supplementary Information:**

The online version contains supplementary material available at 10.1186/s13195-023-01377-5.

## Introduction

Alzheimer’s disease (AD) is an irreversible neurodegenerative disorder characterized clinically by progressive deterioration of memory, behavioral disturbances, and cognitive deficits. The main pathological hallmarks include widespread senile amyloid plaques as well as neurofibrillary tangles, which start decades before the onset of AD [1, 2]. In addition, brain atrophy detected on MRI not only has been reported to be relevant to cognitive decline, but also has been used to reflect the severity of AD [3]. The current strategies for preventing AD onset or delaying AD progression focus on accurately identifying high-risk AD patients among the non-demented population, with a lack of reliable early markers for AD [4]. Evidence from clinical and experimental studies supports the involvement of neuroinflammatory alterations in the preclinical phase of AD, suggesting that anti-inflammatory therapies are potentially promising new directions for the treatment and prevention of dementia [5]. Complement system has been widely recognized as a central system of innate immune defense and a potent driver of neuroinflammation. Its over-activation, dysregulation, or impairment contributes to the pathogenesis of certain autoimmune neurological diseases, which may even lead to neurodegenerative disorders [6]. Accordingly, there is an imperative need to explore whether complement proteins are involved in the progression of AD and their potential as specific markers for early diagnosis of AD.

The complement system consists of more than 40 proteins, and it can be activated by classical, alternative, and lectin pathways depending on the target ligand [7]. Pathological evidence from numerous animal models showed that the complement system has associations with amyloid-β deposition, tau accumulation, and structural brain atrophy [8–11]. The participation of complement proteins in the formation of amyloid plaques in AD patients has been identified in an autopsy report [12]. Previous genome-wide association studies (GWAS) also found the contribution of complements to AD onset [13]. Additionally, it has been demonstrated that C1q, a subcomponent of the first complement component (C1), is responsible for synaptic pruning and synaptic plasticity [14]. The result from a cohort study indicated that complement 3 (C3) and factor H (FH) in cerebrospinal fluid (CSF) were associated with changes in cognition and brain structures [15]. In addition, previous studies have shown that inflammation may contribute to cognitive decline by affecting brain morphology [8, 16]. However, the underlying associations between complement proteins and cognitive decline need to be further explained by in vivo biological and structural evidence, particularly those studies examining whether the AD core pathology or brain structure modulated the association between complement dysregulation and cognitive decline. Nevertheless, the mechanisms underlying the associations between complement proteins and cognitive decline need to be further explained by in vivo biological and structural evidence, especially whether the association is modulated by AD core pathology or brain structure. Given the complexity and size of the complement system, there have been few population-based studies on the utility of CSF complement proteins to date. In the classical complement activation pathway, the recognition of antigen by C1q initiates the process, with C2 being involved. On the other hand, CFB is specifically involved in the alternative pathway. Additionally, C5, C6, and C8 are crucial subcomponents of the membrane attack complex (MAC), which plays a significant role in the downstream cascade of the complement activation pathway. These proteins represent different activation pathways and provide comprehensive information. Herein, we systematically investigated the relationships of CSF complement proteins (C1q, C2, C5, C6, complement component C8 beta chain [C8B], and complement factor B [CFB]) with cognition, AD pathology, and brain structure from the AD Neuroimaging Initiative (ADNI) cohort, and further explored the potential mechanisms underlying the associations between CSF complement proteins and cognition.

## Methods

### Participants

The data used in our study were obtained from the ADNI database, which was launched in 2003 as a multisite longitudinal biomarker research program and was designed to test whether various clinical, biologic, and neuropsychological markers and serial magnetic resonance imaging can be combined to identify the progression of mild cognitive impairment (MCI) and early AD [17].

The study population consisted of 125 MCI participants and 85 cognitively normal (CN) controls. They provided information on complement proteins (C1q, C2, C5, C6, C8B, and CFB) and clinical features at baseline, and data on AD pathology, cognition or brain structures during the follow-up. The subjects were classified into CN (CDR = 0, MMSE > 24) and MCI (CDR = 0.5, MMSE > 24) according to predefined criteria [18]. The follow-up period was 6.07 ± 4.00 years in average (range 1–14 years) for all enrolled participants. Written informed consent signed by all participating individuals or authorized representatives was acquired before protocol-specific procedures were carried out.

### Measurements of CSF AD biomarkers and complement proteins

In ADNI, 16 protein fragments of six complement proteins (C1q, C2, C5, C6, C8B, and CFB) were measured by multiple reaction monitoring (MRM) targeted mass spectrometry [19, 20]. Detailed information on complement proteins assessment and quality control is available at (http://adni.loni.usc.edu/data-samples/biospecimen-data/). The CSF complement proteins data used in our study were log quantified values instead of original protein concentrations, which aimed to avoid biologically significant differences in the levels of two peptides from the same protein that may result from alternative splicing or post-translational modifications. All peptides mapped to the same complement protein were combined into a composite score if they were highly correlated with* r* > 0.5, and uncorrelated fragments were omitted (Additional file 1: Figure S1) [21]. In addition, the concentrations of amyloid beta 42 (Aβ42), total tau (t-tau), and phosphorylated tau (p-tau) in CSF were estimated using a complex xMAP platform (Luminex Corporation) with research-use-only innogenetics (INNO-BIA AlzBio3; Ghent, Belgium) immune assay kit-based reagents at the ADNI Biomarker Core Laboratory (University of Pennsylvania). Study participants from ADNI with available CSF biomarkers data at baseline were grouped according to the CSF levels of AD biomarkers. Precisely, abnormal (A +) or normal (A −) statuses of amyloid pathology were defined by a cutoff value of 976.6 pg/mL for CSF Aβ42 [2] also tau pathological abnormal (T +) or normal (T-) statuses were defined by a cutoff value of 21.8 pg/mL for CSF p-tau (http://adni.loni.usc.edu/methods) [2, 22].

### Cognition

Global and domain-specific cognitive functions were measured by multiple scales. The Mini-Mental State Examination (MMSE), the Clinical Dementia Rating Sum of Boxes (CDRSB), and the Alzheimer Disease Assessment Scale13-item Cognitive subscale (ADAS-Cog13) were applied to evaluate the global cognitive function. Domain-specific cognitive functions were assessed by the neuropsychological test batteries with composite scores to indicate memory (ADNI_MEM), language (ADNI_LAN), and executive function (ADNI_EF) [23–25]. All assessments were performed at baseline and follow-up.

### Neuroimaging

Structural brain magnetic resonance imaging (MRI) was obtained through a Siemens Trio 3.0 T or Vision 1.5-T imaging system. The image processing frameworks Free-surfer software (version 4.3 and 5.1) were applied to estimate the regional volume based on MRI images [26]. The whole brain volume and several regional brain volumes (ventricles, hippocampus, middle temporal lobe, fusiform gyrus, entorhinal cortex) were utilized in our analyses.

### Statistical analysis

The demographical characteristics of the participants in this study are included in Table 1. Categorical variables were shown as percentages using the chi-square test. As for continuous variables, the variables with a normal distribution (Kolmogorov–Smirnov test > 0.05) were reported as mean ± standard deviation (SD), while the non-normally distributed were reported as median (interquartile range, IQR).

**Table 1:** Clinical characteristics of participants in the current study

Characteristics	CN (*n* = 85)	MCI (*n* = 125)	*p* value
Age (years)	75.02 ± 5.19	72.97 ± 6.93	0.163
Gender (F/M)	41/44	42/83	0.033
Education (years)	16 [14 to 18]	16 [14 to 18]	0.371
*APOE-ε4* allele (0/1/2)	64/19/2	56/52/17	** < 0.001**
Lifestyles			
BMI (kg/m^2^)	27.67 [24.13 to 30.55]	27.24 [24.01 to 29.00]	0.067
Smoking habit (Y/N)	28/57	44/81	0.735
Drinking habit (Y/N)	6/79	6/119	0.489
Medical comorbidities			
Hypertension (Y/N)	37/48	53/72	0.871
Diabetes (Y/N)	5/80	11/114	0.434
CSF AD biomarkers			
Aβ42 (pg/ml)	998.65 [746.03 to 1344]	644.9 [520.8 to 844.4]	** < 0.001**
p-tau (pg/ml)	18.90 [15. to 24.85]	31.15 [21.53 to 40.39]	** < 0.001**
t-tau (pg/ml)	213.35 [180.57 to 264]	309.2 [228.6 to 398.4]	** < 0.001**
MRI measures			
Ventricles (mm^3^)	34,199 [21,962 to 44,439]	40,552 [28,917 to 51,791]	**0.001**
Hippocampus (mm^3^)	7347 ± 852.58	6315.47 ± 1084.2	** < 0.001**
Whole brain (mm^3^)	1,025,046.08 ± 99,790.16	1,016,577.32 ± 105,727.25	0.862
Entorhinal (mm^3^)	3857.40 ± 698.18	3263.14 ± 711.18	** < 0.001**
Fusiform (mm^3^)	17,687.60 ± 2280.65	16,610.46 ± 2250.84	0.233
Mid temporal (mm^3^)	20,105.98 ± 2984.73	18,740.46 ± 2808.12	0.059
ICV (mm^3^)	1,554,710.96 ± 171,007.77	1,596,813.67 ± 165,193.37	**0.011**
Cognitive composite measures			
MMSE	29 [29 to 30]	27 [26 to 28]	** < 0.001**
CDRSB	0 [0,0]	1.5 [1 to 2]	** < 0.001**
ADAS-13	9.57 ± 4.33	19.08 ± 6.34	** < 0.001**
ADNI_MEM	0.71 [0.51 to 1.12]	− 0.20 [− 0.42 to 0.03]	** < 0.001**
ADNI_LAN	0.66 [0.37 to 1.04]	0.26 [− 0.07 to 0.60]	** < 0.001**
ADNI_EF	0.69 ± 0.44	0.18 ± 0.50	** < 0.001**
CSF complement			
C1q	34.6819 ± 0.91947	34.7456 ± 0.87754	0.758
C2	50.258 ± 1.38452	49.6265 ± 5.7224	0.130
C5	51.5824 ± 2.16207	51.6726 ± 2.25498	0.647
C6	31.9926 ± 1.26408	31.9713 ± 1.76171	0.581
C8B	45.6711 ± 5.71109	46.2476 ± 3.2088	0.163
CFB	55.1891 ± 1.50536	55.1602 ± 1.6624	0.382

Categorical variables are reported as numbers and percentages; continuous variables are reported as means ± SDs or median [first quartile to third quartile] as appropriate

*Abbreviations: ADAS-13* Alzheimer’s disease Assessment Scale-13, *ADNI* Alzheimer’s Disease Neuroimaging Initiative, *APOE-ε4 Apolipoprotein E4*, *Aβ42* Amyloid β peptide 42, *BMI* Body mass index, *C1q* Complement C1q subcomponent subunit B, *C2* Complement C2, *C5* Complement C5, *C6* Complement C6, *C8B* Complement C8 beta chain, *CDRSB* Clinical Dementia Rating Sum of Boxes, *CFB* Complement factor B, *CN* Normal controls, *CSF* Cerebrospinal fluid, *EF* Executive function, *F* Female, *LAN* Language, *M* Male, *ICV* Intracranial volume, *MEM* Memory function, *MCI* Mild cognitive impairment, *MMSE* Mini-Mental State Examination, *MRI* Magnetic resonance imaging, *p-tau* Phosphorylated tau, *t-tau* Total tau

First, to explore whether there were changes in CSF complement protein concentrations in non-demented individuals, the differences in complement protein concentrations among different groups stratified by cognitive status and pathological status were assessed by *t*-test or one-way ANOVA analyses.

Next, mixed-effect linear models were conducted to assess the longitudinal associations of CSF complement proteins (independent variables) with cognitive function, AD pathology, and brain structural measures in different clinical diagnostic groups. After eliminating the extreme values 3SD above or below the means, all dependent variables were transformed through the “car” package in the R software according to the Box-Cox method to achieve approximate normal distribution. Besides, all variables were standardized by *z*-scale to facilitate comparisons. The following two sensitivity analyses were further carried out to examine the robustness of our main findings: (1) repeating primary results in samples excluding CSF hemoglobin > 1500 ng/ml (10 CN and 18 MCI), as previous literature has reported that CSF blood contamination significantly affects the concentration of certain CSF proteins [27]; (2) additionally adjusting for potential influencing factors, including body mass index, diabetes mellitus, hypertension, the history of alcohol intake or smoking, and the level of CSF C-reaction protein (CRP) [28]. Furthermore, interaction terms between complement proteins with gender, age, and *APOE-ε4* status were utilized to ascertain whether the associations of CSF complement proteins with longitudinal change of cognitive performance were independent of gender, age as well as *APOE-ε4* status.

Finally, we conducted mediation analyses to investigate the potential mechanisms of the associations between CSF complement proteins with global and domain-specific cognitive functions. We utilized regional brain structures as mediators to analyze their mediating effects on the associations between CSF complement proteins and cognition [8, 16]. Linear regression models were fitted in accordance with the methods advanced by Baron and Kenny [29]. To obtain robust results, all mediational exams were analyzed by R software in the “mediate,” “car,” and “lm” packages for 10,000 bootstrap replications.

Covariates in all regression analyses included gender, age, *APOE-ε4* allele (*APOE-ε4*^−/−^  = 0, *APOE-ε4*^−/^^+^ = 1, or *APOE-ε4*^+/+^  = 2), as well as education level, with additional adjustment for intracranial volume when the causal variable was a measurement of brain structure unless otherwise specified. The statistical significance in this study was set at a two-tailed *p* value < 0.05. The Bonferroni method was adopted for the multiple comparisons of cognition, AD pathology, and MRI imaging measurements. All statistical analyses and figure preparation were performed using the R software version 3.5.1 and IBM SPSS Statistics 26.

## Results

### Characteristics of participants

Table 1 summarizes the baseline demographic characteristics, clinical profile, and biological and imaging features of the enrolled ADNI participants. The proportion of females in the study population was 39.5%, and the average baseline age was 73.78 (± 6.36) years. As expected, the cognitively impaired individuals had poorer cognitive performance (i.e., MMSE, CDRSB, ADAS-13, ADNI_MEM, ADNI_EF, and ADNI_LAN, all *p* < 0.001) and higher pathological burden (i.e., Aβ42, p-tau and t-tau, *p* < 0.001) compared with cognitively normal subjects. Besides, MCI individuals had worsening brain atrophy indicated by a larger ventricular volume (*p* = 0.001) as well as smaller hippocampal (*p* < 0.001) and entorhinal volumes (*p* < 0.001). However, there were no significant differences in gender, age, and educational level between the two groups.

### Levels of CSF complement proteins under different diagnostic and pathological statuses

**Fig. 1:**
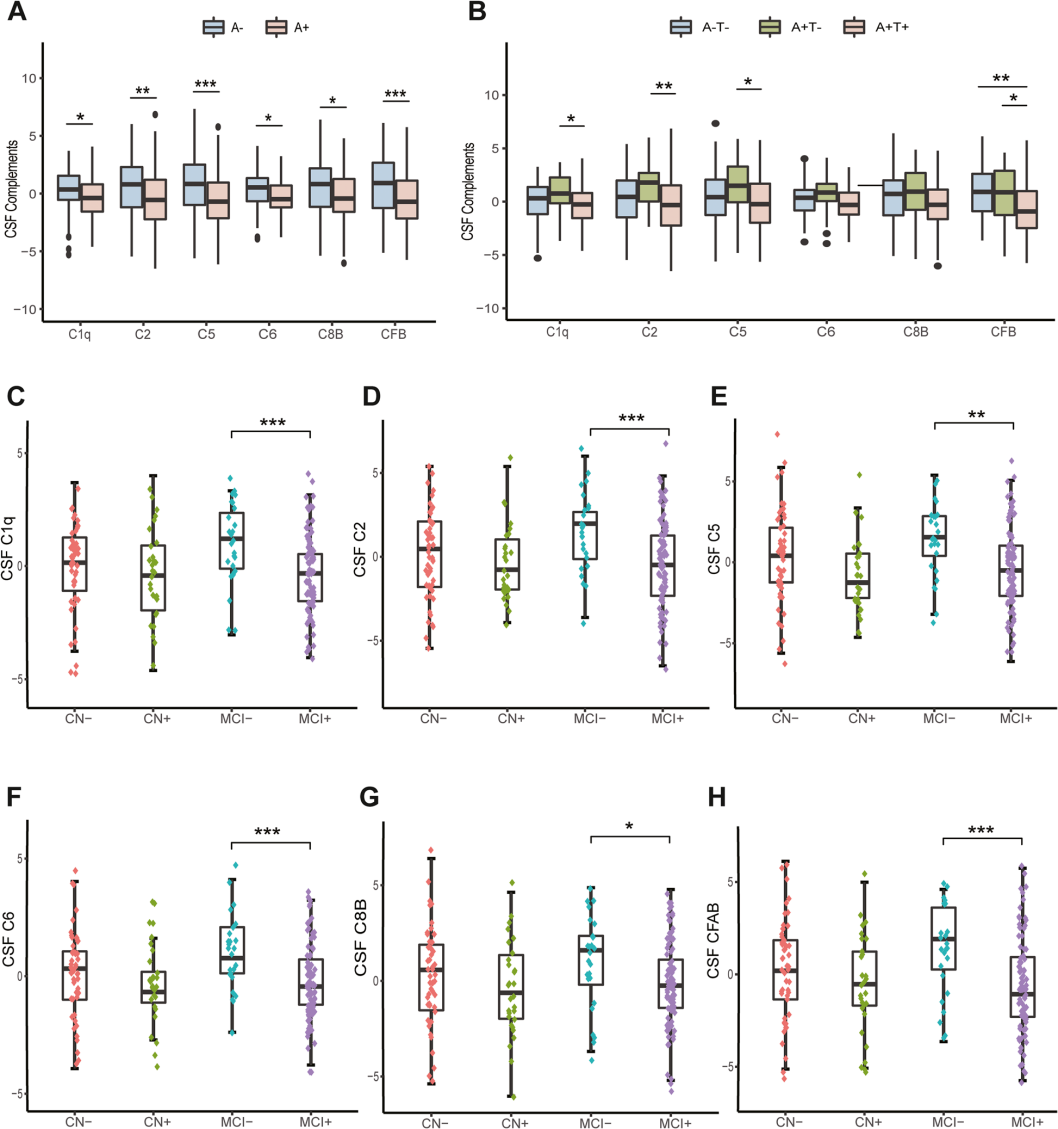
CSF complement protein boxplots for classification groups combining cognitive and pathological status. Boxplots illustrating the log-transformed and *z*-scaled for all complement protein data in cerebrospinal fluid. **A** CSF complement proteins score in A + /A − subgroups. **B** CSF complement proteins score in A − T − /A + T − /A + T + subgroups. **C**–**H** CSF complement protein score in the diagnostic groups stratified by A + /A − status. A − , Aβ42 negative; A + , Aβ42 positive; C1q, complement C1q subcomponent subunit B; C2, complement C2; C5, complement C5; C6, complement C6; C8B, complement component C8 beta chain; CFB, complement factor B; CSF, cerebrospinal fluid; T − , p-tau negative; T + , *p*-tau positive. *p < 0.05, **p < 0.01, and ***p < 0.001

As demonstrated in Table 1, no statistically significant difference was detected in CSF complement protein levels between MCI subjects and CN participants. We further evaluated the relationships of alterations in CSF complement proteins with Aβ deposition and the downstream processes of the amyloid cascade. The individuals falling in the category of A + had significantly lower levels of C1q, C2, C5, C6, C8B, and CFB than the A − subjects (Fig. 1A). And Aβ + MCI participants had significantly lower levels of C1q, C2, C5, C6, C8B, and CFB than Aβ − MCI subjects (Fig. 1C–F). Besides, the ANCOVA analyses revealed that the CSF levels of several complement proteins were also significantly different between the A − T − , A + T − , and A + T + groups. To be specific, the A + T + group had the significantly lowest CSF C1q, C2, C5, and CFB levels compared to A + T − (all *p* < 0.05), but only had the significantly lowest CSF CFB levels (*p* < 0.001) compared to A − T − groups (Fig. 1B).

### Associations of CSF complement proteins with cognition, AD pathology and brain structures

**Fig. 2:**
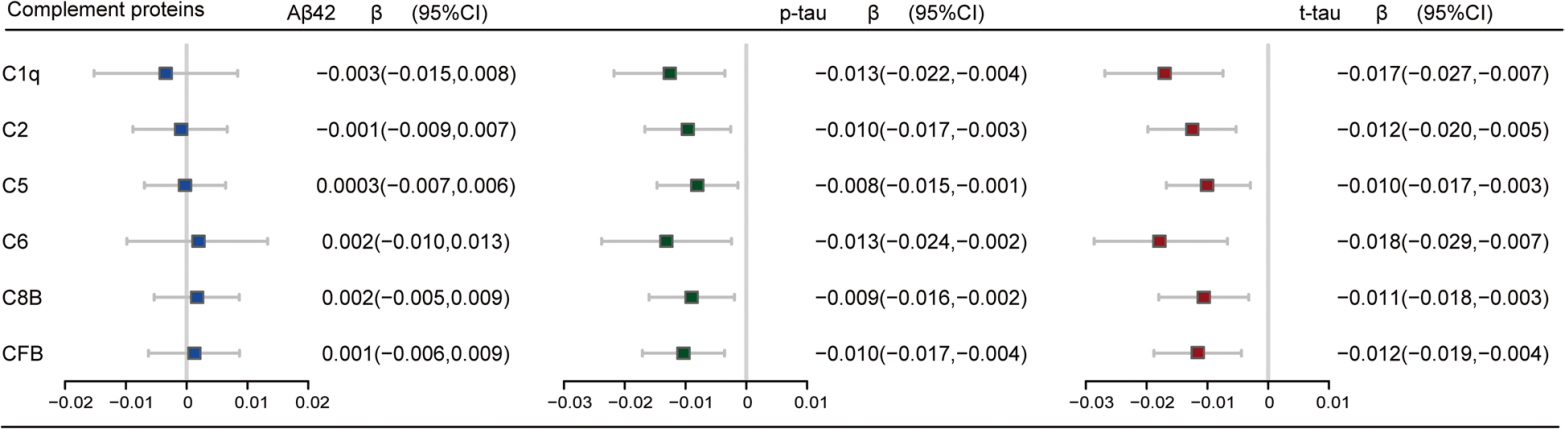
Associations between baseline CSF complement proteins and longitudinal changes of CSF AD biomarkers among CN participants. The mixed effect linear models were applied adjusting for gender, age, educational level, and *APOE-ε4* status. Abbreviations: *APOE*, apolipoprotein E; Aβ42, amyloid β peptide 42; C1q, complement C1q subcomponent subunit B; C2, complement C2; C5, complement C5; C6, complement C6; C8B, complement component C8 beta chain; CFB, complement factor B; CN, cognitively normal; CSF, cerebrospinal fluid; p-tau, phosphorylated tau, t-tau, total tau

**Fig. 3:**
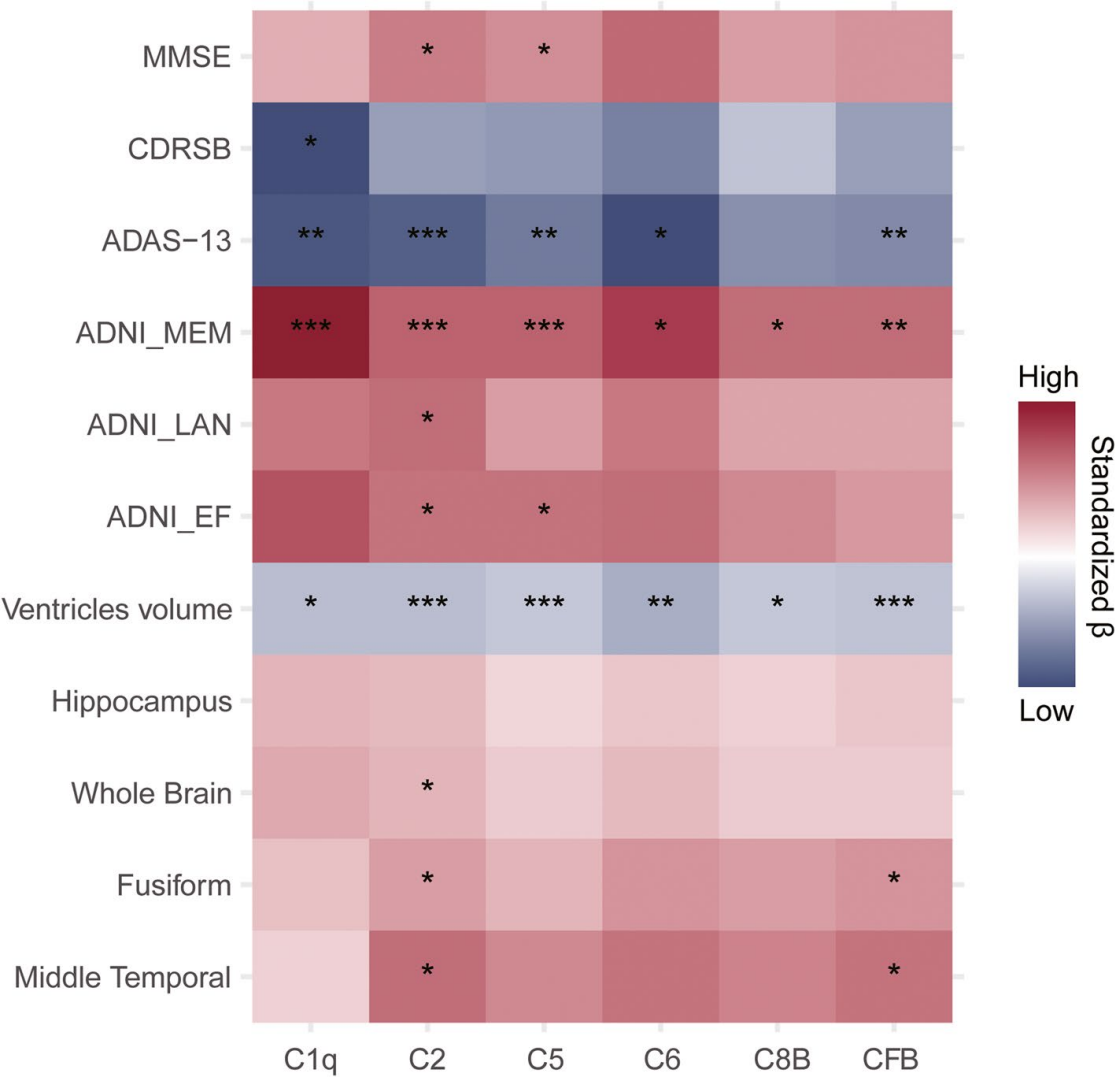
Associations between CSF complement proteins with cognitive and neuroimaging measures in MCI participants. The figure shows the relationships of each baseline CSF complement protein with longitudinal changes in cognitive and neuroimaging measurements. The mixed effect linear models were applied adjusting for gender, age, educational level, *APOE-ε4* status, and additional adjustment for intracranial volume when the causal variable was a measurement of brain structure. Significant results are marked with asterisks. *p < 0.05, **p < 0.01, and ***p < 0.001. ADAS-13, Alzheimer’s disease Assessment Scale-13; ADNI, Alzheimer’s disease Neuroimaging Initiative; C1q, complement C1q subcomponent subunit B; C2, complement C2; C5, complement C5; C6, complement C6; C8B, complement component C8 beta chain; CFB, complement factor B; CDRSB, Clinical Dementia Rating Sum of Boxes; CSF, cerebrospinal fluid; EF, executive function; LAN, language; MEM, memory function; MCI, mild cognitive impairment; MMSE, Mini-Mental State Examination

As shown in Fig. 2 and Additional file 1: Table S1, the mixed effect linear analysis found that among CN subjects, lower CSF complement protein levels were associated with faster elevation of p-tau (C1q: *β* =  − 0.013, *p* = 0.028; C2: β =  − 0.010, *p* = 0.023; C8B: *β* =  − 0.009, *p* = 0.049; CFB: *β* =  − 0.010, *p* = 0.014) and t-tau (C1q: *β* =  − 0.017, *p* = 0.004; C2: *β* =  − 0.012, *p* = 0.005; C5: =  − 0.010, *p* = 0.025; C6: *β* =  − 0.018, *p* = 0.009; C8B: *β* =  − 0.011, *p* = 0.025; CFB: *β* =  − 0.012, *p* = 0.010) levels, whereas no association of CSF complement protein levels with changes in cognition and brain structures during follow-up. In the MCI subjects, higher CSF complement protein levels were correlated with a slower cognitive decline as indicated by the changes in MMSE score (C2: *β* = 0.023, *p* = 0.011; C5: β = 0.020, *p* = 0.042), CDRSB score (C1q: *β* =  − 0.035, *p* = 0.013), ADAS-13 score (C1q: *β* =  − 0.033, *p* = 0.007; C2: *β* =  − 0.031, *p* < 0.001; C5: *β* =  − 0.025, *p* = 0.002; C6: *β* =  − 0.035, *p* = 0.015; CFB: *β* =  − 0.022, *p* = 0.008), ADNI_MEM score (C1q: *β* = 0.042, *p* < 0.001; C2: *β* = 0.028, *p* < 0.001; C5: *β* = 0.028, *p* < 0.001; C6: *β* = 0.035, *p* = 0.029; C8B: *β* = 0.026, *p* = 0.023; CFB: *β* = 0.026, *p* = 0.004), ADNI_LAN score (C2: *β* = 0.026, *p* = 0.010), and ADNI_EF score (C2: *β* = 0.025, *p* = 0.010; C5: *β* = 0.025, *p* = 0.013). Besides, lower CSF complement protein levels were associated with faster ventricular volume expansion (C1q: *β* =  − 0.012, *p* = 0.026; C2: *β* =  − 0.012, *p* < 0.001; C5: *β* =  − 0.010, *p* = 0.001; C6: *β* =  − 0.015, *p* = 0.004; C8B: *β* =  − 0.010, *p* = 0.016; CFB: *β* =  − 0.011, *p* < 0.001), as well as faster atrophy of the whole brain (C2: *β* = 0.013, *p* = 0.025), fusiform (C2: *β* = 0.017, *p* = 0.045; CFB: *β* = 0.019, *p* = 0.017), and middle temporal lobe (C2: *β* = 0.026, *p* = 0.015; CFB: *β* = 0.025, *p* = 0.022) (Fig. 3 and Additional file 1: Table S2). Sensitivity analyses excluding samples with CSF hemoglobin levels > 1500 ng/ml showed that among CN participants, low levels of CSF C1q, C2, and CFB were obviously associated with faster-elevated levels of t-tau rather than p-tau (Additional file 1: Table S5-6). Sensitivity analyses adjusting for all potential influencing factors showed that the identified associations of CSF complement proteins with cognition, AD pathology, and brain structures barely changed, and only the non-significant associations of CSF C2 and C5 with aggravated tau pathology in the MCI group turned significant (Additional file 1: Table S7-8).

Linear regression analyses were performed to better understand if CSF complement protein levels are influenced by demographic factors. As shown in Additional file 1: Table S10, the main effects from age and gender were observed. However, our effect modification analyses showed no significant interactions of CSF complement proteins with them (*p* for interaction > 0.05, Additional file 1: Table S3-4), suggesting that the associations of CSF complement protein levels with longitudinal change of cognitive performance were independent of gender, age, and *APOE-ε4* status.

### Causal mediation analyses

Preliminary analyses in different cognitive groups demonstrated that the reduced CSF complement peptides C1q, C2, C5, and CFB were associated with longitudinal brain atrophy (i.e., ventricles, whole brain, middle temporal lobe, or fusiform gyrus) and cognitive decline in MCI participants. Accordingly, we conducted mediation analyses in MCI subjects to examine whether the associations between CSF complement proteins and longitudinal cognitive changes were mediated by neuroimaging markers.

**Fig. 4:**
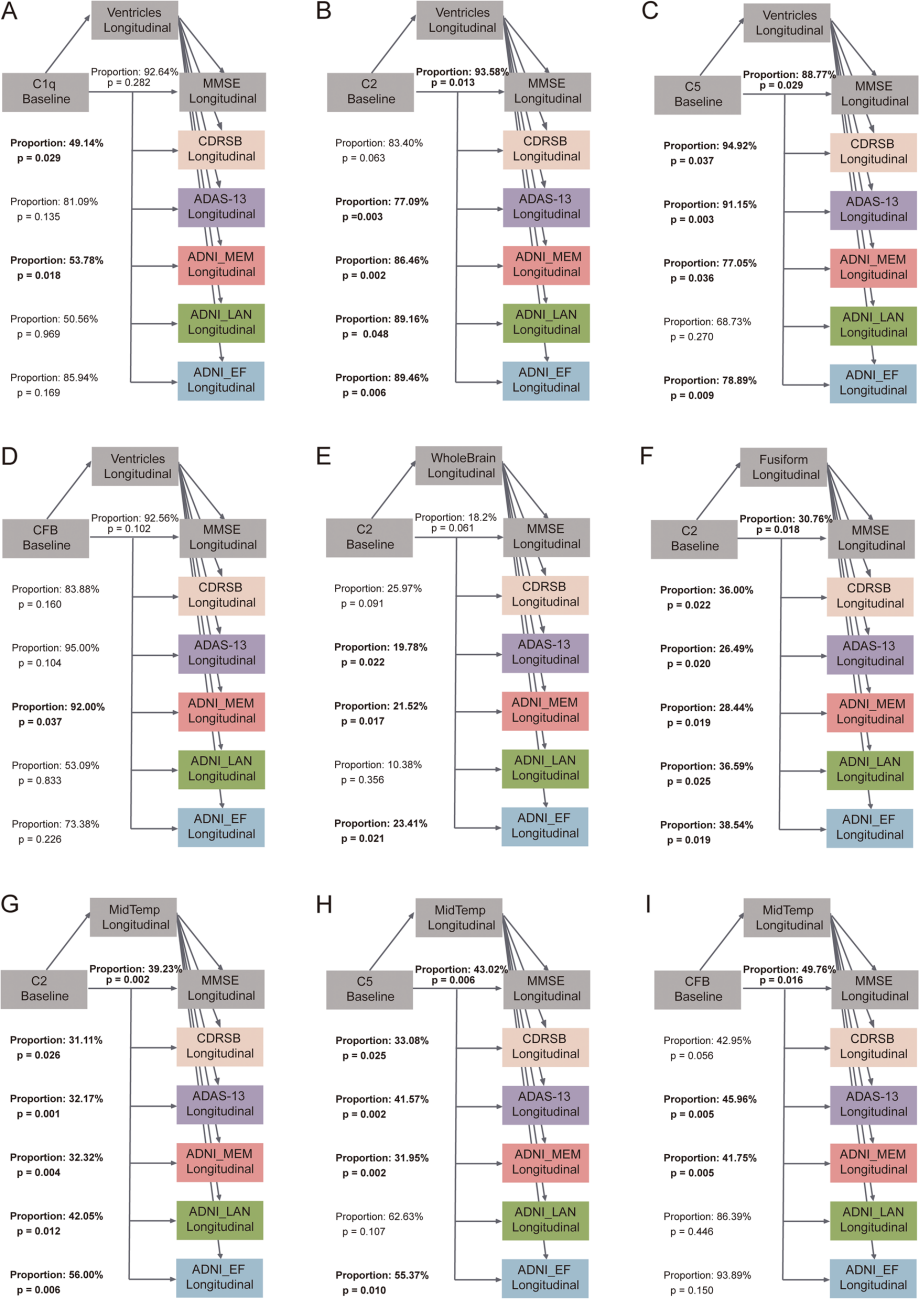
Explanatory analyses of associations between CSF complement proteins, brain structures, and cognition in MCI participants. The relationship between CSF complement proteins and cognition was mediated by ventricles (**A**–**D**), whole brain (**E**), fusiform gyrus (**F**), or middle temporal lobe (**G**–**I**). The proportions shown in the figure indicate the proportion of mediating factors in the total effect of brain structures on cognition. ADAS-13, Alzheimer’s Disease Assessment Scale-13; ADNI, Alzheimer’s Disease Neuroimaging Initiative; CDRSB, Clinical Dementia Rating Sum of Boxes; EF, executive function; LAN, language; MCI, mild cognitive impairment; MEM, memory function; MMSE, Mini-Mental State Examination

As illustrated in Fig. 4 and Additional file 1: Table S9, the mediation models showed statistically significant indirect and total effects after controlling for gender, age, education, and *APOE-ε4* status. Our analyses suggested that the regional brain structures showed at least a trend to significance in the mediation path, with the mediating proportions ranging from 19.78 to 94.92%. The direct impact of reduced CSF complement peptides C1q, C2, C5, and CFB on cognitive decline was also observed in the MCI participants ( Additional file 1: Table S2). Precisely, we found the relationships between CSF complement proteins and cognitive performance were mediated by ventricles (CSF C1q, 49.14 to 53.78% of total effect; CSF C2, 77.09 to 93.58% of total effect; CSF C5, 77.05 to 94.92% of total effect; and CSF CFB, 92.00% of total effect, Fig. 4A–D), whole brain (CSF C2, 19.78 to 23.42% of total effect, Fig. 4E), fusiform gyrus (CSF C2, 26.49 to 38.54% of total effect, Fig. 4F), and middle temporal lobe (CSF C2, 31.11 to 56.00% of total effect; CSF C5, 31.95 to 53.37% of total effect; and CSF CFB, 41.75 to 49.76% of total effect, Fig. 4G–I). Besides, additional analysis of CSF clusterin yielded similar results (Additional file 1: Table S11-12).

## Discussion

This study systematically investigated the association of CSF complement proteins (C1q, C2, C5, C6, C8B, and CFB) with cognitive decline, AD pathology and brain structures, as well as the mediation effects of regional brain structures on cognition. Several key conclusions were drawn: (1) Subjects with low levels of complement proteins in CSF have more severe evolution of AD pathologies. Precisely, the CSF complement protein levels were obviously lower in the A + individuals than in A − individuals and the A + T + group had significantly lower levels of CSF complement proteins compared to A + T − and A − T − groups; (2) lower complement protein levels might contribute to faster cognitive decline and atrophy of multiple brain regions in the MCI population; (3) mediation studies showed that whole brain volume, regional ventricular, middle temporal, or fusiform volume mediated the association between low levels of CSF complement proteins and accelerated cognitive decline. These findings suggest low CSF complement protein levels accelerate the progression of AD and provide a plausible pathway to link neuroinflammation with cognitive decline.

Previous studies in genomics [30] and proteomics cohort studies [31, 32], meta-analysis [33], and autopsy cases [34] have been used to investigate the potential plasma and CSF complement components as biomarkers for differentiating between AD and non-AD groups and predicting the transition from MCI to AD. However, these studies have often yielded conflicting or inconsistent results. In this study, we focused on examining the diagnostic ability of complement proteins in CSF during the preclinical stage of AD. No significant differences in CSF complement protein levels between diagnostic groups measured by the MMSE (MCI versus CN) were revealed, suggesting CSF complement proteins could not serve as diagnostic biomarkers in the early stages of AD. Congruously, a previous study conducted on a non-demented population demonstrated the suitability of CSF C3 and CFH (measured by MRM) levels as biomarkers for early diagnosis of AD is insufficient [15]. Our findings added new evidence in the current field, because the MS analysis can accurately and sensitively measure the levels of various types of complement proteins in CSF.

Our research has provided further evidence supporting the link between low levels of CSF complement proteins and increased Aβ accumulation, although we did not observe a significant linear correlation. Furthermore, our study revealed a positive longitudinal association between CSF complement protein levels and tau pathology in the CN population. Moreover, our previous research about CSF clusterin also suggested the early involvement of CSF complement proteins in AD progression. It can be deduced that in the pathological phase of amyloid, the levels of CSF complement proteins are reduced with Aβ. Our research indicated the levels of CSF complement proteins changed after the alterations of markers for brain amyloidosis and downstream tau pathology. C1q and C4BP have been shown to bind to the Aβ peptide in the AD brain, and the reduction in cerebrospinal fluid complement levels is thought to be a result of plaque trapping [11, 35]. Thus, we can infer the capture of complement proteins (not only C1q and C4BP) in plaques may cause a decrease in CSF levels of these proteins. The meaningless linear relationship suggests that the reduction in cerebrospinal fluid levels of complement proteins cannot be readily explained by such a simple model as mentioned above. Further animal experiments are warranted to be conducted in the future. The role of complement as a driver of inflammation in the AD brain was first proposed in the 1980s [36]. Evidence from genetics, clinical studies, and animal models has been confusing and inconsistent in recent decades, but the widely accepted view is that dysregulation of the balance between complement activation and inhibition is responsible for neuroinflammatory and neurodegenerative diseases. In vitro experiments have shown that C1q enhanced neuronal survival and exerted neuroprotective effects against certain toxic substances and upregulated in early AD [37]. C3 inhibition accelerated the deposition of Aβ plaques and increased neurodegeneration in mouse models of AD [38, 39]. On the contrary, C1q has also been proven to be required for amyloid β-associated synaptic toxicity in mouse models [40]. One study found complement-dependent pathways and microglia, which were inappropriately activated, mediated early synaptic loss in AD [41], and another study found the inhibition of C1q impaired classical complement cascade processes, thereby reducing glial activation and synaptic loss [42]. Moreover, genetic analyses have suggested that C1R mutations may contribute to the progression of AD by regulating the accumulation of Aβ [43, 44]. However, it is noted that not all studies have found significant correlations between variations in CLU and CR and CSF Aβ and tau [45, 46]. These discrepancies may be attributed to the fact that most mouse models simulate early-onset AD; the mutations found in these models are single-gene mutations rather than the common multi-gene mutations seen in late-onset AD. Consequently, the findings from these mouse models may only provide limited insights and cannot be generalized to all cases. A cross-sectional study using ELISA found an association between CSF C3 and CSF p-tau [40], whereas another population-based study reported no significant association between CSF C3 and Aβ42, p-tau, or t-tau, but a positive correlation with Aβ40 [47]. Many complement components and functions exhibit age- and sex-related variation. Differences in age, sex, sample size, and disease states seem to be responsible for the discrepancies among the published studies. The advanced experimental models that better mimic the late-onset AD and large-scale cohorts using the MRM platform (having high reproducibility within laboratories, across laboratories, and across different instrument platforms) are warranted to validate our findings.

The association of lower levels of CSF complement protein with poor cognitive function and decreased regional brain volumes was observed in our study. Numerous studies investigating these associations yielded disparate results. Our findings have been further corroborated by a study using an enzyme-linked immunosorbent assay to estimate CSF C1q levels. The study showed a strong correlation of reduced C1q levels and decreased overall measures of mental status and specific measures of cognitive function (e.g., word recall, word recognition, delayed recall, and memory tasks) [31]. Another cohort study employing the Luminex assay has reported a similar association of low levels of CSF C3 with faster cognitive decline. Additionally, this study demonstrated the role of lower FH levels in contributing to brain atrophy, specifically larger lateral ventricular volumes, in individuals with mild cognitive impairment (MCI) [15]. Furthermore, the acute and chronic animal models of Alzheimer’s disease also showed that CSF complement inhibits glial hyperactivation, neuroinflammation, and cognitive decline [30]. Carriers of the CFH risk allele have increased internal olfactory thickness at younger ages and higher rates of atrophy as the disease progresses, which suggests that the CFH risk allele may play a reversed role in the early and late stages of AD [48]. In contrast, a population-based study using Luminex assay analysis showed an opposite relationship between CSF C3 and FH and cognitive performance [49]; an autopsy study revealed that CSF C3 levels were significantly related to MMSE scores in AD subjects, whereas they were not significantly correlated in the MCI [50]. Our study supports a strong link between complement system proteins and cognitive decline in Alzheimer’s disease, and these findings provide evidence for clinical complement-targeted therapies for cognitive decline.

The modification effects of age and gender on CSF complement were observed in this study, which is consistent with the majority of previous findings [11, 47, 49]. In line with our finding, a study based on the ADNI database has shown that the *APOE-ε4* status is not the main factor influencing the levels of complement proteins [15]. However, preliminary clinical studies illustrated inconsistent findings. An increase in *APOE-ε4* copy number has been reported to be inversely related to the level of complement proteins in cerebrospinal fluid [51]. The interaction between complement C3 and *APOE-ε4* leads to increased Alzheimer’s disease-related pathology [40]. Both cohort studies encompassed healthy populations with AD, MCI, and cognitively normal healthy populations. The difference in findings may be due to the different populations included in the studies. To our knowledge, this is the first study to explore the modification effects by age, sex, and *APOE-ε4 status on the* relationship between cerebrospinal fluid complement and cognitive performance in CN and MCI populations, respectively, although we did not find positive results. The study based on a larger population and covering different stages of AD is needed to confirm our findings. Besides, our study found that neuroimaging markers might mediate the associations between CSF complement proteins (C1q, C2, C5, CFB and clusterin) and cognitive decline. In line with our findings, it has been reported that global and regional gray matter volumes mediate the association between inflammation and cognitive decline [16]. This suggests that the observed difference in neuroimaging markers may help prevent complement-related cognitive deficits. Our mediation analyses confirmed the presence of mediated factors for complement-related cognitive deficits, including whole brain volume, ventricular volume, fusiform, and middle temporal volume. Our findings put forward new insights into the action of complement system in the development and progression of AD.

The strengths of our study include the use of subjects with detailed clinical and neuropsychological testing, cerebrospinal fluid marker measurements, longitudinal follow-up data, available *APOE-ε4* genotype information, and hemoglobin data to control blood contamination. After adjusting for a wide range of confounders, the sensitivity analyses supported that the results were robust. In addition, our study utilized a mass spectrometry-based method for complement protein detection. MRM (Multiple Reaction Monitoring) is the most sensitive mass spectrometry platform, allowing for specific and sensitive quantification of a large number of peptides and proteins in biological samples within a single run. Compared to previous ELISA, 2D gel electrophoresis methods, we obtained reliable data on a wider variety of complement species. It has been demonstrated to have high reproducibility within laboratories, between laboratories, and across different instrument platforms [19]. MRM is a reliable method for validating and testing hypotheses along the translational pathway of biomarkers.

The present study also has several limitations. First, the generalizability of our results may be restricted by the source of the study population, the insufficient number of AD patients to cover the entire course of AD, and the absence of longitudinal data on CSF complement proteins information. Second, the CSF complement protein data used in our study were log quantified values instead of original protein concentrations, so the range of differences for this protein in this study cannot be used as a practical clinical reference. Third, the types of complement proteins detectable in the ADNI database are limited, so we cannot conduct a more comprehensive analysis. It is noted that the reason why CSF complement C3 and C4 were not addressed in this study is because our research team is currently using ADNI proteomics data to study their role in AD (not yet published). Besides, supplementary analyses on CSF clusterin have validated the robustness and credibility of our conclusions. In addition, our results should be replicated in larger longitudinal cohorts as well as in animal experiments to increase credibility. Fourth, despite all efforts to control for confounding factors, the remaining confounders cannot be completely ignored. For example, regarding potential confounding effects of medication, our study did not exclude the use of CNS active drugs, such as narcotic analgesics or psychostimulants with anticholinergic properties, antidepressants, anti-Parkinson drugs, etc.

Taken together, our findings suggested that CSF complement proteins might be prognostic biomarkers for accelerated cognitive decline, although it needs to be validated in an independent cohort. Furthermore, these findings make a contribution to our understanding of the inflammatory neurobiology of cognitive function. Finally, further investigations in the future are warranted to explore mechanisms of complement system activation, including the role of classical and alternative cascade processes in the development of AD.

### Supplementary Information

**Additional file 1: Figure S1.** Correlation matrix of complement proteins and their peptides. **Table S1.** Associations of CSF complement proteins with longitudinal cognitive function, AD pathology and neuroimaging in cognitively normal participants. **Table S2.** Associations of CSF complement proteins with longitudinal cognitive function, AD pathology and neuroimaging in mild cognitive impairment participants. **Table S3.** Interactions effects of CSF complement proteins on longitudinal change of cognitive function in cognitively normal participants. **Table S4.** Interactions effects of CSF complement proteins on longitudinal change of cognitive function in mild cognitive impairment participants. **Table S5.** Sensitivity analyses of CSF complement proteins with cognitive function, AD pathology and neuroimaging in cognitively normal participants limitied with CSF haemoglobin. **Table S6.** Sensitivity analyses of CSF complement proteins with cognitive function, AD pathology and neuroimaging in mild cognitive impairment participants limitied with CSF haemoglobin. **Table S7.** Sensitivity analyses of CSF complement proteins with cognitive function, AD pathology and neuroimaging in cognitively normal participants adjusted for full models. **Table S8.** Sensitivity analyses of CSF complement proteins with cognitive function, AD pathology and neuroimaging in mild cognitive impairment participants adjusted for full models. **Table S9.** Mediating effects of regional brain structures on the association between CSF complement proteins and cognition. **Table S10.** Association of CSF complement biomarkers with age, gender and APOE-ε4 presence. **Table S11.** Associations of CSF clusterin with longitudinal cognitive function, AD pathology and neuroimaging in cognitively normal and mild cognitive impairment participants. **Table S12.** Mediating effects of regional brain structures on the association between CSF clusterin and cognition in mild cognitive impairment participants.

## Data Availability

The datasets used and/or analyzed during the current study are available from the corresponding author upon reasonable request.
