# Supplementary material for: Associations of cerebrospinal fluid complement proteins with Alzheimer’s pathology, cognition, and brain structure in non-dementia elderly
Source: Alzheimers Res Ther. 2024 Jan 18;16:12. doi: 10.1186/s13195-023-01377-5 (PMC10795368; doi:10.1186/s13195-023-01377-5)
Supplement: Supplementary file 1 — Additional file 1: Figure S1. Correlation matrix of complement proteins and their peptides. Table S1. Associations of CSF complement proteins with longitudinal cognitive function, AD pathology and neuroimaging in cognitively normal participants. Table S2. Associations of CSF complement proteins with longitudinal cognitive function, AD pathology and neuroimaging in mild cognitive impairment participants. Table S3. Interactions effects of CSF complement proteins on longitudinal change of cognitive function in cognitively normal participants. Table S4. Interactions effects of CSF complement proteins on longitudinal change of cognitive function in mild cognitive impairment participants. Table S5. Sensitivity analyses of CSF complement proteins with cognitive function, AD pathology and neuroimaging in cognitively normal participants limitied with CSF haemoglobin. Table S6. Sensitivity analyses of CSF complement proteins with cognitive function, AD pathology and neuroimaging in mild cognitive impairment participants limitied with CSF haemoglobin. Table S7. Sensitivity analyses of CSF complement proteins with cognitive function, AD pathology and neuroimaging in cognitively normal participants adjusted for full models. Table S8. Sensitivity analyses of CSF complement proteins with cognitive function, AD pathology and neuroimaging in mild cognitive impairment participants adjusted for full models. Table S9. Mediating effects of regional brain structures on the association between CSF complement proteins and cognition. Table S10. Association of CSF complement biomarkers with age, gender and APOE-ε4 presence. Table S11. Associations of CSF clusterin with longitudinal cognitive function, AD pathology and neuroimaging in cognitively normal and mild cognitive impairment participants. Table S12. Mediating effects of regional brain structures on the association between CSF clusterin and cognition in mild cognitive impairment participants. [file 13195_2023_1377_MOESM1_ESM.doc]

**Associations of cerebrospinal fluid complement proteins with Alzheimer’s pathology, cognitive performance and brain structure in individuals without dementia**

Supplementary material

**Content**

[Figure S1 Correlation matrix of complement proteins and their peptides. 1](#__RefHeading___Toc14035)

[Table S1 Associations of CSF complement proteins with longitudinal cognitive function, AD pathology and neuroimaging in cognitively normal participants. 2](#__RefHeading___Toc14860)

[Table S2 Associations of CSF complement proteins with longitudinal cognitive function, AD pathology and neuroimaging in mild cognitive impairment participants. 4](#__RefHeading___Toc15158)

[Table S3 Interactions effects of CSF complement proteins on longitudinal change of cognitive function in cognitively normal participants. 6](#__RefHeading___Toc27113)

[Table S4 Interactions effects of CSF complement proteins on longitudinal change of cognitive function in mild cognitive impairment participants. 8](#__RefHeading___Toc3884)

[Table S5 Sensitivity analyses of CSF complement proteins with cognitive function, AD pathology and neuroimaging in cognitively normal participants limitied with CSF haemoglobin. 10](#__RefHeading___Toc29567)

[Table S6 Sensitivity analyses of CSF complement proteins with cognitive function, AD pathology and neuroimaging in mild cognitive impairment participants limitied with CSF haemoglobin. 12](#__RefHeading___Toc20900)

[Table S7 Sensitivity analyses of CSF complement proteins with cognitive function, AD pathology and neuroimaging in cognitively normal participants adjusted for full models. 14](#__RefHeading___Toc16263)

[Table S8 Sensitivity analyses of CSF complement proteins with cognitive function, AD pathology and neuroimaging in mild cognitive impairment participants adjusted for full models. 16](#__RefHeading___Toc8147)

[Table S9 Mediating effects of regional brain structures on the association between CSF complement proteins and cognition. 17](#__RefHeading___Toc30789)

[Table S10 Association of CSF complement biomarkers with age, gender and *APOE*-ε4 presence. 20](#__RefHeading___Toc23906)

[Table S11 Associations of CSF clusterin with longitudinal cognitive function, AD pathology and neuroimaging in cognitively normal and mild cognitive impairment participants. 21](#__RefHeading___Toc4957)

[Table S12 Mediating effects of regional brain structures on the association between CSF clusterin and cognition in mild cognitive impairment participants. 22](#__RefHeading___Toc28517)

## Figure S1 Correlation matrix of complement proteins and their peptides.


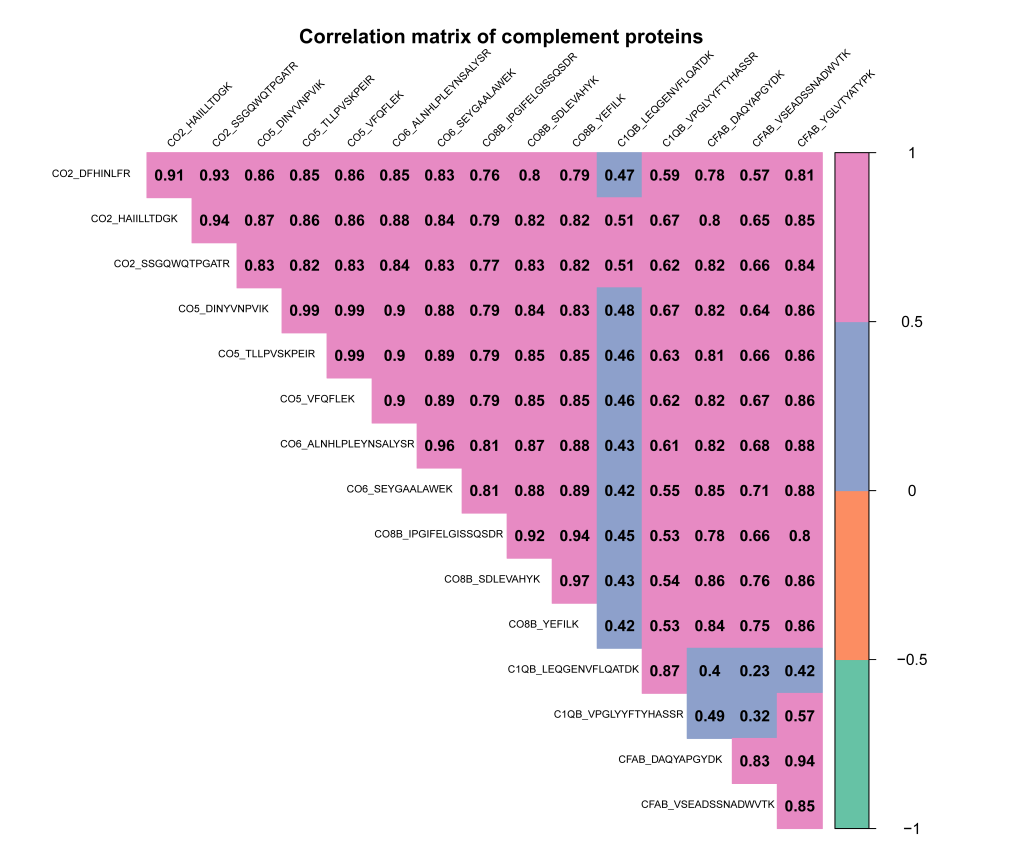

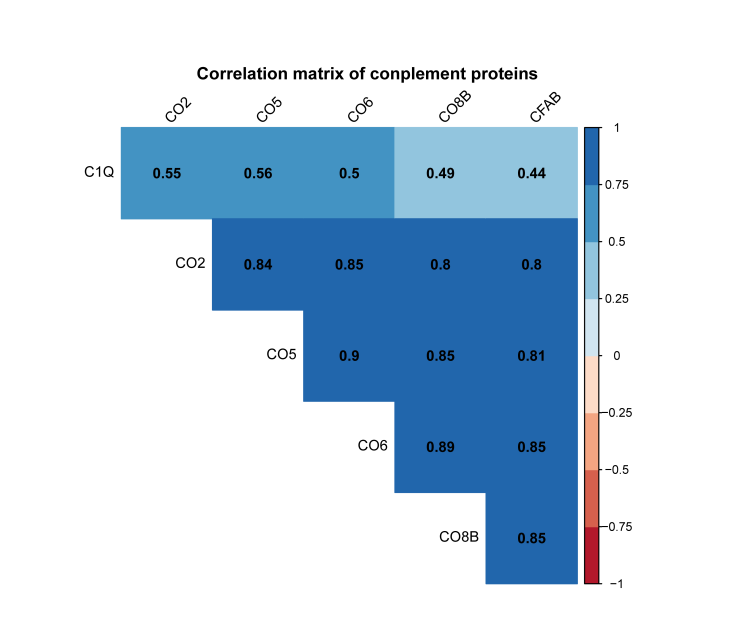


Peptides of the same complement protein class were strongly correlated(A)(0.81< r< 0.99, p < 0.001). As for the data of complement protein scores, all correlations were calculated (B)(0.44< r< 0.99, p < 0.001). C1q, complement C1q subcomponent subunit B; C2, complement C2; C5, complement C5; C6, complement C6; C8B, complement component C8 beta chain; CFB, complement factor B.

## Table S1 Associations of CSF complement proteins with longitudinal cognitive function, AD pathology and neuroimaging in cognitively normal participants.

| Variables | C1q | | | C2 | | | C5 | | | C6 | | | C8B | | | CFB | | |
| --- | --- | --- | --- | --- | --- | --- | --- | --- | --- | --- | --- | --- | --- | --- | --- | --- | --- | --- |
|  | β | p value | adjust-p value | β | p value | adjust-p value | β | p value | adjust-p value | β | p value | adjust-p value | β | p value | adjust-p value | β | p value | adjust-p value |
| MMSE | -0.011 | 0.166 | 0.995 | -0.002 | 0.793 | 1.000 | 0.000 | 0.967 | 1.000 | 0.001 | 0.923 | 1.000 | 0.003 | 0.610 | 1.000 | 0.004 | 0.550 | 1.000 |
| CDRSB | 0.016 | 0.056 | 0.335 | 0.006 | 0.404 | 1.000 | 0.002 | 0.759 | 1.000 | 0.001 | 0.962 | 1.000 | -0.004 | 0.531 | 1.000 | 0.000 | 0.943 | 1.000 |
| ADAS-13 | 0.001 | 0.986 | 1.000 | -0.006 | 0.286 | 1.000 | -0.006 | 0.185 | 1.000 | -0.009 | 0.238 | 1.000 | -0.006 | 0.248 | 1.000 | -0.008 | 0.106 | 0.637 |
| ADNI_MEM | 0.001 | 0.865 | 1.000 | 0.002 | 0.757 | 1.000 | 0.001 | 0.848 | 1.000 | -0.002 | 0.824 | 1.000 | -0.002 | 0.737 | 1.000 | 0.004 | 0.555 | 1.000 |
| ADNI_LAN | -0.003 | 0.750 | 1.000 | -0.003 | 0.675 | 1.000 | 0.002 | 0.712 | 1.000 | 0.009 | 0.347 | 1.000 | 0.003 | 0.614 | 1.000 | 0.001 | 0.862 | 1.000 |
| ADNI_EF | 0.004 | 0.647 | 1.000 | 0.011 | 0.121 | 0.727 | 0.013 | 0.047 | 0.280 | 0.018 | 0.092 | 0.549 | 0.012 | 0.103 | 0.620 | 0.011 | 0.095 | 0.572 |
| Aβ | -0.003 | 0.569 | 1.000 | -0.001 | 0.816 | 1.000 | 0.000 | 0.935 | 1.000 | 0.002 | 0.739 | 1.000 | 0.002 | 0.628 | 1.000 | 0.001 | 0.735 | 1.000 |
| p-tau | -0.013 | 0.009 | 0.028 | -0.010 | 0.011 | 0.032 | -0.008 | 0.022 | 0.066 | -0.013 | 0.020 | 0.060 | -0.009 | 0.016 | 0.049 | -0.010 | 0.005 | 0.014 |
| t-tau | -0.017 | 0.001 | 0.004 | -0.012 | 0.002 | 0.005 | -0.010 | 0.008 | 0.025 | -0.018 | 0.003 | 0.009 | -0.011 | 0.008 | 0.025 | -0.012 | 0.003 | 0.010 |
| Ventricular | -0.001 | 0.605 | 1.000 | -0.001 | 0.750 | 1.000 | -0.001 | 0.594 | 1.000 | 0.000 | 0.915 | 1.000 | -0.002 | 0.374 | 1.000 | -0.001 | 0.764 | 1.000 |
| Hippocampus | 0.001 | 0.962 | 1.000 | 0.002 | 0.547 | 1.000 | 0.003 | 0.417 | 1.000 | 0.004 | 0.449 | 1.000 | 0.005 | 0.235 | 1.000 | 0.003 | 0.364 | 1.000 |
| Whole brain | 0.001 | 0.885 | 1.000 | 0.001 | 0.696 | 1.000 | 0.002 | 0.590 | 1.000 | 0.002 | 0.627 | 1.000 | 0.006 | 0.088 | 0.530 | 0.002 | 0.460 | 1.000 |
| Entorhinal | -0.005 | 0.369 | 1.000 | -0.006 | 0.233 | 1.000 | -0.002 | 0.672 | 1.000 | -0.003 | 0.678 | 1.000 | 0.002 | 0.746 | 1.000 | -0.004 | 0.442 | 1.000 |
| Fusiform | 0.008 | 0.148 | 0.889 | 0.003 | 0.472 | 1.000 | 0.004 | 0.275 | 1.000 | 0.005 | 0.477 | 1.000 | 0.007 | 0.104 | 0.625 | 0.000 | 0.904 | 1.000 |
| Middle temporal | 0.003 | 0.588 | 1.000 | 0.003 | 0.431 | 1.000 | 0.003 | 0.437 | 1.000 | 0.007 | 0.299 | 1.000 | 0.007 | 0.083 | 0.500 | 0.005 | 0.188 | 1.000 |

Age, gender, education and *ApoE-ε4* carrier status adjusted mixed effect models. Adjusted p values are corrected for multiple comparisons (Bonferroni). ADAS-13 Alzheimer’ s disease Assessment Scale-13, ADNI, Alzheimer’s disease Neuroimaging Initiative, *APOE-ε4* apolipoprotein E4, Aβ42, amyloid β peptide 42, C1q, complement C1q subcomponent subunit B; C2, complement C2; C5, complement C5; C6, complement C6; C8B, complement component C8 beta chain; CFB, complement factor B; CDRSB, Clinical Dementia Rating Sum of Boxes; CSF, cerebrospinal fuid; EF, executive function; LAN, language; MEM, memory function; MMSE, Mini-Mental State Examination;p-tau phosphorylated tau, t-tau total tau.

## Table S2 Associations of CSF complement proteins with longitudinal cognitive function, AD pathology and neuroimaging in mild cognitive impairment participants.

| Variables | C1q | | | C2 | | | C5 | | | C6 | | | C8B | | | CFB | | |
| --- | --- | --- | --- | --- | --- | --- | --- | --- | --- | --- | --- | --- | --- | --- | --- | --- | --- | --- |
|  | β | p value | adjust-p value | β | p value | adjust-p value | β | p value | adjust-p value | β | p value | adjust-p value | β | p value | adjust-p value | β | p value | adjust-p value |
| MMSE | 0.014 | 0.197 | 1.000 | 0.023 | 0.002 | 0.011 | 0.020 | 0.007 | 0.042 | 0.027 | 0.032 | 0.190 | 0.017 | 0.048 | 0.285 | 0.019 | 0.014 | 0.081 |
| CDRSB | -0.035 | 0.002 | 0.013 | -0.018 | 0.019 | 0.114 | -0.019 | 0.010 | 0.057 | -0.024 | 0.054 | 0.322 | -0.011 | 0.196 | 1.000 | -0.018 | 0.018 | 0.107 |
| ADAS-13 | -0.033 | 0.001 | 0.007 | -0.031 | <0.001 | <0.0001 | -0.025 | <0.001 | 0.002 | -0.035 | 0.003 | 0.015 | -0.021 | 0.009 | 0.052 | -0.022 | 0.001 | 0.008 |
| ADNI_MEM | 0.042 | <0.001 | 0.001 | 0.028 | <0.001 | 0.001 | 0.028 | <0.001 | 0.001 | 0.035 | 0.005 | 0.029 | 0.026 | 0.004 | 0.023 | 0.026 | 0.001 | 0.004 |
| ADNI_LAN | 0.024 | 0.054 | 0.326 | 0.026 | 0.002 | 0.010 | 0.017 | 0.043 | 0.255 | 0.024 | 0.090 | 0.541 | 0.016 | 0.109 | 0.656 | 0.016 | 0.066 | 0.396 |
| ADNI_EF | 0.031 | 0.009 | 0.056 | 0.025 | 0.002 | 0.010 | 0.025 | 0.002 | 0.013 | 0.026 | 0.050 | 0.297 | 0.021 | 0.029 | 0.173 | 0.018 | 0.024 | 0.142 |
| Aβ | 0.010 | 0.293 | 0.878 | 0.005 | 0.424 | 1.000 | 0.005 | 0.427 | 1.000 | 0.011 | 0.277 | 0.830 | 0.005 | 0.441 | 1.000 | 0.007 | 0.285 | 0.854 |
| p-tau | -0.006 | 0.313 | 0.938 | -0.005 | 0.218 | 0.655 | -0.007 | 0.072 | 0.215 | -0.009 | 0.155 | 0.465 | -0.008 | 0.078 | 0.235 | -0.006 | 0.119 | 0.358 |
| t-tau | -0.010 | 0.166 | 0.499 | -0.009 | 0.072 | 0.217 | -0.010 | 0.036 | 0.109 | -0.012 | 0.135 | 0.404 | -0.013 | 0.021 | 0.062 | -0.009 | 0.060 | 0.181 |
| Ventricular | -0.012 | 0.004 | 0.026 | -0.012 | <0.001 | <0.001 | -0.010 | <0.001 | 0.001 | -0.015 | 0.001 | 0.004 | -0.010 | 0.003 | 0.016 | -0.011 | <0.001 | 0.000 |
| Hippocampus | 0.013 | 0.082 | 0.493 | 0.012 | 0.017 | 0.100 | 0.007 | 0.177 | 1.000 | 0.010 | 0.215 | 1.000 | 0.008 | 0.142 | 0.851 | 0.010 | 0.037 | 0.225 |
| Whole brain | 0.015 | 0.030 | 0.178 | 0.013 | 0.004 | 0.025 | 0.009 | 0.048 | 0.287 | 0.012 | 0.114 | 0.686 | 0.009 | 0.076 | 0.455 | 0.009 | 0.054 | 0.324 |
| Entorhinal | 0.011 | 0.214 | 1.000 | 0.002 | 0.763 | 1.000 | 0.005 | 0.447 | 1.000 | 0.005 | 0.608 | 1.000 | 0.003 | 0.705 | 1.000 | 0.004 | 0.498 | 1.000 |
| Fusiform | 0.011 | 0.248 | 1.000 | 0.017 | 0.008 | 0.045 | 0.013 | 0.049 | 0.291 | 0.019 | 0.077 | 0.464 | 0.017 | 0.024 | 0.145 | 0.019 | 0.003 | 0.017 |
| Middle temporal | 0.008 | 0.576 | 1.000 | 0.026 | 0.003 | 0.015 | 0.021 | 0.020 | 0.121 | 0.025 | 0.093 | 0.556 | 0.022 | 0.031 | 0.185 | 0.025 | 0.004 | 0.022 |

Age, gender, education and *ApoE-ε4* carrier status adjusted multiple linear model. Adjusted p values are corrected for multiple comparisons (Bonferroni). ADAS-13 Alzheimer’ s disease Assessment Scale-13, ADNI, Alzheimer’s disease Neuroimaging Initiative, *APOE-ε4* apolipoprotein E4, Aβ42, amyloid β peptide 42, C1q, complement C1q subcomponent subunit B; C2, complement C2; C5, complement C5; C6, complement C6; C8B, complement component C8 beta chain; CFB, complement factor B; CDRSB, Clinical Dementia Rating Sum of Boxes; CSF, cerebrospinal fuid; EF, executive function; LAN, language; MEM, memory function; MMSE, Mini-Mental State Examination;p-tau phosphorylated tau, t-tau total tau.

## Table S3 Interactions effects of CSF complement proteins on longitudinal change of cognitive function in cognitively normal participants.

| Variables | C1q | | | C2 | | | C5 | | | C6 | | | C8B | | | CFB | | |
| --- | --- | --- | --- | --- | --- | --- | --- | --- | --- | --- | --- | --- | --- | --- | --- | --- | --- | --- |
|  | β | p value | adjust-p value | β | p value | adjust-p value | β | p value | adjust-p value | β | p value | adjust-p value | β | p value | adjust-p value | β | p value | adjust-p value |
| × Age |  |  |  |  |  |  |  |  |  |  |  |  |  |  |  |  |  |  |
| MMSE | 0.011 | 0.220 | 1.000 | -0.002 | 0.820 | 1.000 | -0.004 | 0.600 | 1.000 | -0.005 | 0.655 | 1.000 | -0.001 | 0.904 | 1.000 | -0.005 | 0.521 | 1.000 |
| CDRSB | -0.011 | 0.261 | 1.000 | -0.001 | 0.883 | 1.000 | 0.002 | 0.790 | 1.000 | 0.0002 | 0.988 | 1.000 | 0.002 | 0.822 | 1.000 | 0.007 | 0.464 | 1.000 |
| ADAS | -0.014 | 0.155 | 0.929 | -0.003 | 0.677 | 1.000 | 0.002 | 0.737 | 1.000 | 0.001 | 0.899 | 1.000 | 0.001 | 0.905 | 1.000 | 0.011 | 0.219 | 1.000 |
| ADNI_MEM | 0.007 | 0.456 | 1.000 | -0.0002 | 0.982 | 1.000 | -0.004 | 0.563 | 1.000 | -0.004 | 0.723 | 1.000 | -0.006 | 0.544 | 1.000 | -0.015 | 0.100 | 0.601 |
| ADNI_LAN | 0.016 | 0.107 | 0.642 | 0.004 | 0.621 | 1.000 | 0.008 | 0.288 | 1.000 | 0.009 | 0.430 | 1.000 | 0.012 | 0.201 | 1.000 | 0.004 | 0.701 | 1.000 |
| ADNI_EF | -0.004 | 0.721 | 1.000 | -0.003 | 0.665 | 1.000 | -0.004 | 0.630 | 1.000 | -0.003 | 0.765 | 1.000 | -0.005 | 0.608 | 1.000 | -0.005 | 0.592 | 1.000 |
| × Gender |  |  |  |  |  |  |  |  |  |  |  |  |  |  |  |  |  |  |
| MMSE | 0.047 | 0.682 | 1.000 | 0.069 | 0.434 | 1.000 | 0.059 | 0.484 | 1.000 | 0.087 | 0.475 | 1.000 | 0.070 | 0.429 | 1.000 | 0.021 | 0.794 | 1.000 |
| CDRSB | -0.035 | 0.773 | 1.000 | -0.048 | 0.606 | 1.000 | -0.059 | 0.502 | 1.000 | -0.124 | 0.329 | 1.000 | -0.044 | 0.629 | 1.000 | -0.081 | 0.348 | 1.000 |
| ADAS | 0.038 | 0.752 | 1.000 | -0.046 | 0.620 | 1.000 | -0.038 | 0.664 | 1.000 | -0.143 | 0.255 | 1.000 | -0.069 | 0.450 | 1.000 | -0.041 | 0.633 | 1.000 |
| ADNI_MEM | -0.114 | 0.347 | 1.000 | 0.026 | 0.783 | 1.000 | 0.076 | 0.394 | 1.000 | 0.149 | 0.243 | 1.000 | 0.077 | 0.401 | 1.000 | 0.051 | 0.552 | 1.000 |
| ADNI_LAN | -0.051 | 0.683 | 1.000 | -0.136 | 0.154 | 0.924 | -0.189 | 0.035 | 0.211 | -0.152 | 0.245 | 1.000 | -0.085 | 0.367 | 1.000 | -0.128 | 0.145 | 0.870 |
| ADNI_EF | -0.103 | 0.396 | 1.000 | -0.110 | 0.235 | 1.000 | -0.102 | 0.242 | 1.000 | -0.055 | 0.669 | 1.000 | -0.017 | 0.849 | 1.000 | -0.026 | 0.761 | 1.000 |
| × *APOE* |  |  |  |  |  |  |  |  |  |  |  |  |  |  |  |  |  |  |
| MMSE | 0.083 | 0.524 | 1.000 | -0.043 | 0.694 | 1.000 | -0.058 | 0.541 | 1.000 | -0.084 | 0.547 | 1.000 | -0.008 | 0.950 | 1.000 | -0.021 | 0.831 | 1.000 |
| CDRSB | -0.007 | 0.962 | 1.000 | -0.038 | 0.746 | 1.000 | 0.023 | 0.818 | 1.000 | 0.083 | 0.573 | 1.000 | -0.007 | 0.956 | 1.000 | -0.017 | 0.871 | 1.000 |
| ADAS | -0.008 | 0.956 | 1.000 | 0.022 | 0.849 | 1.000 | 0.066 | 0.504 | 1.000 | 0.075 | 0.606 | 1.000 | -0.008 | 0.951 | 1.000 | 0.003 | 0.977 | 1.000 |
| ADNI_MEM | 0.039 | 0.780 | 1.000 | 0.007 | 0.950 | 1.000 | -0.018 | 0.855 | 1.000 | -0.060 | 0.685 | 1.000 | 0.026 | 0.845 | 1.000 | 0.028 | 0.788 | 1.000 |
| ADNI_LAN | -0.230 | 0.110 | 0.660 | -0.049 | 0.685 | 1.000 | 0.014 | 0.895 | 1.000 | 0.077 | 0.613 | 1.000 | 0.083 | 0.540 | 1.000 | 0.108 | 0.323 | 1.000 |
| ADNI_EF | -0.291 | 0.039 | 0.232 | 0.059 | 0.617 | 1.000 | 0.061 | 0.543 | 1.000 | 0.190 | 0.200 | 1.000 | 0.195 | 0.134 | 0.806 | 0.246 | 0.019 | 0.112 |

Age, gender, education and *APOE-ε4* carrier status adjusted mixed effect models. Adjusted p values are corrected for multiple comparisons (Bonferroni). ADAS-13 Alzheimer’ s disease Assessment Scale-13, ADNI, Alzheimer’s disease Neuroimaging Initiative, *APOE-ε4* apolipoprotein E4, Aβ42, amyloid β peptide 42, C1q, complement C1q subcomponent subunit B; C2, complement C2; C5, complement C5; C6, complement C6; C8B, complement component C8 beta chain; CFB, complement factor B; CDRSB, Clinical Dementia Rating Sum of Boxes; CSF, cerebrospinal fuid; EF, executive function; LAN, language; MEM, memory function; MMSE, Mini-Mental State Examination;p-tau phosphorylated tau, t-tau total tau.

## Table S4 Interactions effects of CSF complement proteins on longitudinal change of cognitive function in mild cognitive impairment participants.

| Variables | C1q | | | C2 | | | C5 | | | C6 | | | C8B | | | CFB | | |
| --- | --- | --- | --- | --- | --- | --- | --- | --- | --- | --- | --- | --- | --- | --- | --- | --- | --- | --- |
|  | β | p value | adjust-p value | β | p value | adjust-p value | β | p value | adjust-p value | β | p value | adjust-p value | β | p value | adjust-p value | β | p value | adjust-p value |
| × Age |  |  |  |  |  |  |  |  |  |  |  |  |  |  |  |  |  |  |
| MMSE | 0.001 | 0.885 | 1.000 | -0.006 | 0.192 | 1.000 | -0.007 | 0.171 | 1.000 | -0.006 | 0.426 | 1.000 | -0.004 | 0.381 | 1.000 | -0.001 | 0.861 | 1.000 |
| CDRSB | 0.004 | 0.539 | 1.000 | 0.009 | 0.050 | 0.301 | 0.009 | 0.072 | 0.433 | 0.011 | 0.169 | 1.000 | 0.006 | 0.223 | 1.000 | 0.005 | 0.258 | 1.000 |
| ADAS | 0.008 | 0.231 | 1.000 | 0.005 | 0.195 | 1.000 | 0.007 | 0.110 | 0.657 | 0.007 | 0.333 | 1.000 | 0.004 | 0.417 | 1.000 | 0.002 | 0.713 | 1.000 |
| ADNI_MEM | -0.008 | 0.196 | 1.000 | -0.006 | 0.155 | 0.932 | -0.008 | 0.076 | 0.456 | -0.008 | 0.280 | 1.000 | -0.004 | 0.468 | 1.000 | -0.001 | 0.907 | 1.000 |
| ADNI_LAN | 0.0003 | 0.968 | 1.000 | -0.005 | 0.208 | 1.000 | -0.006 | 0.219 | 1.000 | -0.002 | 0.748 | 1.000 | -0.001 | 0.792 | 1.000 | -0.001 | 0.806 | 1.000 |
| ADNI_EF | -0.005 | 0.425 | 1.000 | -0.005 | 0.282 | 1.000 | -0.003 | 0.581 | 1.000 | 0.003 | 0.747 | 1.000 | 0.002 | 0.742 | 1.000 | 0.003 | 0.488 | 1.000 |
| × Gender |  |  |  |  |  |  |  |  |  |  |  |  |  |  |  |  |  |  |
| MMSE | 0.200 | 0.078 | 0.466 | -0.056 | 0.468 | 1.000 | -0.047 | 0.559 | 1.000 | 0.0004 | 0.998 | 1.000 | -0.047 | 0.619 | 1.000 | 0.025 | 0.736 | 1.000 |
| CDRSB | -0.171 | 0.128 | 0.768 | 0.044 | 0.574 | 1.000 | 0.042 | 0.610 | 1.000 | 0.016 | 0.901 | 1.000 | 0.073 | 0.446 | 1.000 | 0.037 | 0.618 | 1.000 |
| ADAS | -0.179 | 0.096 | 0.575 | 0.080 | 0.273 | 1.000 | 0.093 | 0.231 | 1.000 | 0.123 | 0.295 | 1.000 | 0.094 | 0.299 | 1.000 | 0.044 | 0.534 | 1.000 |
| ADNI_MEM | 0.124 | 0.244 | 1.000 | 0.001 | 0.986 | 1.000 | -0.045 | 0.560 | 1.000 | -0.007 | 0.952 | 1.000 | 0.007 | 0.940 | 1.000 | 0.040 | 0.576 | 1.000 |
| ADNI_LAN | 0.216 | 0.052 | 0.310 | -0.080 | 0.294 | 1.000 | 0.002 | 0.981 | 1.000 | 0.012 | 0.924 | 1.000 | -0.023 | 0.801 | 1.000 | -0.008 | 0.917 | 1.000 |
| ADNI_EF | 0.169 | 0.133 | 0.797 | -0.109 | 0.149 | 0.896 | -0.033 | 0.684 | 1.000 | -0.0002 | 0.999 | 1.000 | -0.018 | 0.847 | 1.000 | 0.012 | 0.877 | 1.000 |
| × *APOE* |  |  |  |  |  |  |  |  |  |  |  |  |  |  |  |  |  |  |
| MMSE | 0.128 | 0.204 | 1.000 | -0.015 | 0.815 | 1.000 | -0.001 | 0.983 | 1.000 | -0.082 | 0.470 | 1.000 | -0.105 | 0.181 | 1.000 | -0.061 | 0.353 | 1.000 |
| CDRSB | -0.168 | 0.091 | 0.546 | -0.091 | 0.170 | 1.000 | -0.056 | 0.406 | 1.000 | -0.021 | 0.854 | 1.000 | -0.018 | 0.819 | 1.000 | -0.032 | 0.630 | 1.000 |
| ADAS | -0.231 | 0.014 | 0.087 | 0.00004 | 0.999 | 1.000 | -0.012 | 0.846 | 1.000 | 0.095 | 0.381 | 1.000 | 0.109 | 0.148 | 0.885 | 0.082 | 0.189 | 1.000 |
| ADNI_MEM | 0.175 | 0.062 | 0.373 | 0.025 | 0.695 | 1.000 | 0.033 | 0.609 | 1.000 | -0.021 | 0.844 | 1.000 | -0.053 | 0.480 | 1.000 | -0.056 | 0.377 | 1.000 |
| ADNI_LAN | 0.240 | 0.013 | 0.080 | 0.037 | 0.563 | 1.000 | 0.024 | 0.715 | 1.000 | -0.062 | 0.573 | 1.000 | -0.026 | 0.737 | 1.000 | 0.002 | 0.973 | 1.000 |
| ADNI_EF | 0.066 | 0.506 | 1.000 | 0.002 | 0.979 | 1.000 | -0.009 | 0.889 | 1.000 | -0.038 | 0.739 | 1.000 | -0.036 | 0.643 | 1.000 | -0.011 | 0.862 | 1.000 |

Age, gender, education and *APOE-ε4* carrier status adjusted multiple linear model. Adjusted p values are corrected for multiple comparisons (Bonferroni). ADAS-13 Alzheimer’ s disease Assessment Scale-13, ADNI, Alzheimer’s disease Neuroimaging Initiative, *APOE-ε4* apolipoprotein E4, Aβ42, amyloid β peptide 42, C1q, complement C1q subcomponent subunit B; C2, complement C2; C5, complement C5; C6, complement C6; C8B, complement component C8 beta chain; CFB, complement factor B; CDRSB, Clinical Dementia Rating Sum of Boxes; CSF, cerebrospinal fuid; EF, executive function; LAN, language; MEM, memory function; MMSE, Mini-Mental State Examination;p-tau phosphorylated tau, t-tau total tau.

## Table S5 Sensitivity analyses of CSF complement proteins with cognitive function, AD pathology and neuroimaging in cognitively normal participants limitied with CSF haemoglobin.

| Variables | C1q | | | C2 | | | C5 | | | C6 | | | C8B | | | CFB | | |
| --- | --- | --- | --- | --- | --- | --- | --- | --- | --- | --- | --- | --- | --- | --- | --- | --- | --- | --- |
|  | β | p value | adjust-p value | β | p value | adjust-p value | β | p value | adjust-p value | β | p value | adjust-p value | β | p value | adjust-p value | β | p value | adjust-p value |
| MMSE | -0.017 | 0.064 | 0.386 | -0.006 | 0.436 | 1.000 | -0.004 | 0.555 | 1.000 | -0.004 | 0.720 | 1.000 | 0.001 | 0.923 | 1.000 | 0.001 | 0.894 | 1.000 |
| CDRSB | 0.024 | 0.014 | 0.085 | 0.011 | 0.164 | 0.984 | 0.009 | 0.250 | 1.000 | 0.011 | 0.363 | 1.000 | 0.001 | 0.950 | 1.000 | 0.003 | 0.703 | 1.000 |
| ADAS-13 | 0.002 | 0.778 | 1.000 | -0.007 | 0.205 | 1.000 | -0.009 | 0.115 | 0.692 | -0.011 | 0.213 | 1.000 | -0.008 | 0.219 | 1.000 | -0.009 | 0.111 | 1.000 |
| ADNI_MEM | -0.001 | 0.901 | 1.000 | 0.000 | 0.997 | 1.000 | -0.002 | 0.821 | 1.000 | -0.007 | 0.552 | 1.000 | -0.006 | 0.452 | 1.000 | 0.001 | 0.918 | 1.000 |
| ADNI_LAN | 0.000 | 0.966 | 1.000 | -0.002 | 0.832 | 1.000 | 0.006 | 0.438 | 1.000 | 0.014 | 0.202 | 1.000 | 0.009 | 0.274 | 1.000 | 0.004 | 0.595 | 1.000 |
| ADNI_EF | -0.001 | 0.921 | 1.000 | 0.007 | 0.380 | 0.726 | 0.011 | 0.189 | 0.280 | 0.013 | 0.310 | 1.000 | 0.009 | 0.314 | 1.000 | 0.008 | 0.309 | 1.000 |
| Aβ | -0.012 | 0.101 | 0.304 | -0.007 | 0.122 | 0.366 | -0.007 | 0.133 | 0.399 | -0.007 | 0.352 | 1.000 | -0.003 | 0.468 | 1.000 | -0.003 | 0.530 | 1.000 |
| p-tau | -0.010 | 0.075 | 0.224 | -0.009 | 0.034 | 0.101 | -0.008 | 0.062 | 0.185 | -0.012 | 0.071 | 0.212 | -0.008 | 0.072 | 0.217 | -0.010 | 0.024 | 0.072 |
| t-tau | -0.019 | 0.004 | 0.011 | -0.013 | 0.005 | 0.014 | -0.010 | 0.024 | 0.073 | -0.018 | 0.013 | 0.039 | -0.010 | 0.033 | 0.100 | -0.013 | 0.006 | 0.017 |
| Ventricular | 0.0003 | 0.936 | 1.000 | 0.001 | 0.578 | 1.000 | 0.001 | 0.573 | 1.000 | 0.003 | 0.355 | 1.000 | 0.000 | 0.949 | 1.000 | 0.001 | 0.555 | 1.000 |
| Hippocampus | -0.005 | 0.419 | 1.000 | 0.001 | 0.870 | 1.000 | 0.001 | 0.822 | 1.000 | 0.002 | 0.800 | 1.000 | 0.003 | 0.570 | 1.000 | 0.001 | 0.732 | 1.000 |
| Whole brain | -0.001 | 0.786 | 1.000 | 0.000 | 0.990 | 1.000 | 0.000 | 0.912 | 1.000 | 0.000 | 0.934 | 1.000 | 0.005 | 0.178 | 1.000 | 0.001 | 0.844 | 1.000 |
| Entorhinal | -0.007 | 0.297 | 1.000 | -0.007 | 0.202 | 1.000 | -0.003 | 0.608 | 1.000 | -0.005 | 0.556 | 1.000 | 0.001 | 0.832 | 1.000 | -0.004 | 0.416 | 1.000 |
| Fusiform | 0.008 | 0.259 | 0.888 | 0.002 | 0.739 | 1.000 | 0.003 | 0.584 | 1.000 | 0.001 | 0.886 | 1.000 | 0.006 | 0.291 | 1.000 | -0.002 | 0.686 | 1.000 |
| Middle temporal | 0.000 | 0.962 | 1.000 | 0.002 | 0.674 | 1.000 | 0.001 | 0.800 | 1.000 | 0.004 | 0.583 | 1.000 | 0.007 | 0.185 | 1.000 | 0.006 | 0.234 | 1.000 |

Age, gender, education and *APOE-ε4* carrier status adjusted multiple linear model. Adjusted p values are corrected for multiple comparisons (Bonferroni). ADAS-13 Alzheimer’ s disease Assessment Scale-13, ADNI, Alzheimer’s disease Neuroimaging Initiative, *APOE-ε4* apolipoprotein E4, Aβ42, amyloid β peptide 42, C1q, complement C1q subcomponent subunit B; C2, complement C2; C5, complement C5; C6, complement C6; C8B, complement component C8 beta chain; CFB, complement factor B; CDRSB, Clinical Dementia Rating Sum of Boxes; CSF, cerebrospinal fuid; EF, executive function; LAN, language; MEM, memory function; MMSE, Mini-Mental State Examination;p-tau phosphorylated tau, t-tau total tau.

## Table S6 Sensitivity analyses of CSF complement proteins with cognitive function, AD pathology and neuroimaging in mild cognitive impairment participants limitied with CSF haemoglobin.

| Variables | C1q | | | C2 | | | C5 | | | C6 | | | C8B | | | CFB | | |
| --- | --- | --- | --- | --- | --- | --- | --- | --- | --- | --- | --- | --- | --- | --- | --- | --- | --- | --- |
|  | β | p value | adjust-p value | β | p value | adjust-p value | β | p value | adjust-p value | β | p value | adjust-p value | β | p value | adjust-p value | β | p value | adjust-p value |
| MMSE | 0.013 | 0.286 | 1.000 | 0.022 | 0.005 | 0.029 | 0.019 | 0.016 | 0.096 | 0.023 | 0.089 | 0.535 | 0.014 | 0.115 | 0.690 | 0.017 | 0.036 | 0.213 |
| CDRSB | -0.031 | 0.014 | 0.082 | -0.018 | 0.029 | 0.175 | -0.020 | 0.013 | 0.076 | -0.024 | 0.091 | 0.544 | -0.010 | 0.258 | 1.000 | -0.016 | 0.048 | 0.286 |
| ADAS-13 | -0.030 | 0.008 | 0.050 | -0.032 | <0.001 | <0.001 | -0.025 | 0.001 | 0.003 | -0.036 | 0.005 | 0.029 | -0.021 | 0.015 | 0.087 | -0.021 | 0.005 | 0.033 |
| ADNI_MEM | 0.041 | 0.001 | 0.004 | 0.029 | <0.001 | 0.001 | 0.029 | 0.000 | 0.001 | 0.034 | 0.011 | 0.068 | 0.024 | 0.010 | 0.061 | 0.023 | 0.004 | 0.022 |
| ADNI_LAN | 0.021 | 0.149 | 0.895 | 0.028 | 0.002 | 0.013 | 0.018 | 0.050 | 0.302 | 0.027 | 0.097 | 0.581 | 0.017 | 0.110 | 0.661 | 0.015 | 0.117 | 0.703 |
| ADNI_EF | 0.032 | 0.017 | 0.104 | 0.026 | 0.003 | 0.017 | 0.025 | 0.004 | 0.025 | 0.026 | 0.089 | 0.533 | 0.021 | 0.035 | 0.208 | 0.019 | 0.037 | 0.219 |
| Aβ | 0.013 | 0.232 | 0.696 | 0.001 | 0.855 | 1.000 | 0.001 | 0.924 | 1.000 | 0.000 | 0.982 | 1.000 | -0.001 | 0.933 | 1.000 | 0.001 | 0.852 | 1.000 |
| p-tau | -0.011 | 0.103 | 0.309 | -0.007 | 0.141 | 0.423 | -0.008 | 0.060 | 0.181 | -0.012 | 0.116 | 0.348 | -0.009 | 0.064 | 0.192 | -0.008 | 0.093 | 0.281 |
| t-tau | -0.014 | 0.083 | 0.251 | -0.011 | 0.051 | 0.154 | -0.011 | 0.034 | 0.101 | -0.014 | 0.120 | 0.359 | -0.014 | 0.019 | 0.058 | -0.011 | 0.056 | 0.167 |
| Ventricular | -0.010 | 0.041 | 0.247 | -0.012 | <0.001 | <0.001 | -0.011 | <0.001 | 0.002 | -0.016 | 0.002 | 0.011 | -0.009 | 0.009 | 0.056 | -0.011 | <0.001 | 0.001 |
| Hippocampus | 0.011 | 0.199 | 0.493 | 0.010 | 0.042 | 0.254 | 0.005 | 0.321 | 1.000 | 0.007 | 0.450 | 1.000 | 0.007 | 0.242 | 1.000 | 0.008 | 0.129 | 0.777 |
| Whole brain | 0.011 | 0.192 | 0.178 | 0.013 | 0.012 | 0.074 | 0.009 | 0.073 | 0.435 | 0.011 | 0.215 | 1.000 | 0.009 | 0.134 | 0.806 | 0.008 | 0.111 | 0.666 |
| Entorhinal | 0.007 | 0.459 | 1.000 | 0.003 | 0.682 | 1.000 | 0.006 | 0.305 | 1.000 | 0.008 | 0.419 | 1.000 | 0.004 | 0.519 | 1.000 | 0.006 | 0.355 | 1.000 |
| Fusiform | 0.007 | 0.503 | 1.000 | 0.017 | 0.009 | 0.055 | 0.013 | 0.050 | 0.301 | 0.020 | 0.085 | 0.509 | 0.017 | 0.030 | 0.178 | 0.018 | 0.009 | 0.055 |
| Middle temporal | 0.002 | 0.882 | 1.000 | 0.027 | 0.005 | 0.031 | 0.021 | 0.032 | 0.193 | 0.025 | 0.140 | 0.839 | 0.022 | 0.049 | 0.293 | 0.025 | 0.011 | 0.065 |

Age, gender, education and *APOE-ε4* carrier status adjusted multiple linear model. Adjusted p values are corrected for multiple comparisons (Bonferroni). ADAS-13 Alzheimer’ s disease Assessment Scale-13, ADNI, Alzheimer’s disease Neuroimaging Initiative, *APOE-ε4* apolipoprotein E4, Aβ42, amyloid β peptide 42, C1q, complement C1q subcomponent subunit B; C2, complement C2; C5, complement C5; C6, complement C6; C8B, complement component C8 beta chain; CFB, complement factor B; CDRSB, Clinical Dementia Rating Sum of Boxes; CSF, cerebrospinal fuid; EF, executive function; LAN, language; MEM, memory function; MMSE, Mini-Mental State Examination;p-tau phosphorylated tau, t-tau total tau.

## Table S7 Sensitivity analyses of CSF complement proteins with cognitive function, AD pathology and neuroimaging in cognitively normal participants adjusted for full models.

| Variables | C1q | | | C2 | | | C5 | | | C6 | | | C8B | | | CFB | | |
| --- | --- | --- | --- | --- | --- | --- | --- | --- | --- | --- | --- | --- | --- | --- | --- | --- | --- | --- |
|  | β | p value | adjust-p value | β | p value | adjust-p value | β | p value | adjust-p value | β | p value | adjust-p value | β | p value | adjust-p value | β | p value | adjust-p value |
| MMSE | -0.011 | 0.164 | 0.982 | -0.002 | 0.784 | 1.000 | 0.000 | 0.981 | 1.000 | 0.001 | 0.947 | 1.000 | 0.003 | 0.625 | 1.000 | 0.004 | 0.557 | 1.000 |
| CDRSB | 0.016 | 0.057 | 0.341 | 0.006 | 0.401 | 1.000 | 0.002 | 0.752 | 1.000 | 0.000 | 0.980 | 1.000 | -0.005 | 0.503 | 1.000 | 0.000 | 0.979 | 1.000 |
| ADAS-13 | 0.001 | 0.862 | 1.000 | 0.002 | 0.780 | 1.000 | 0.001 | 0.874 | 1.000 | -0.002 | 0.810 | 1.000 | -0.002 | 0.738 | 1.000 | 0.004 | 0.558 | 1.000 |
| ADNI_MEM | -0.002 | 0.761 | 1.000 | -0.002 | 0.704 | 1.000 | 0.003 | 0.674 | 1.000 | 0.010 | 0.321 | 1.000 | 0.004 | 0.592 | 1.000 | 0.001 | 0.845 | 1.000 |
| ADNI_LAN | 0.000 | 0.981 | 1.000 | -0.006 | 0.271 | 1.000 | -0.007 | 0.172 | 1.000 | -0.010 | 0.227 | 1.000 | -0.006 | 0.242 | 1.000 | -0.008 | 0.103 | 0.617 |
| ADNI_EF | 0.004 | 0.646 | 1.000 | 0.011 | 0.115 | 0.690 | 0.013 | 0.044 | 0.264 | 0.018 | 0.093 | 0.557 | 0.012 | 0.109 | 0.654 | 0.011 | 0.103 | 0.620 |
| Aβ | -0.004 | 0.559 | 1.000 | -0.001 | 0.803 | 1.000 | 0.000 | 0.932 | 1.000 | 0.002 | 0.745 | 1.000 | 0.002 | 0.622 | 1.000 | 0.001 | 0.736 | 1.000 |
| p-tau | -0.012 | 0.012 | 0.036 | -0.009 | 0.014 | 0.043 | -0.008 | 0.027 | 0.082 | -0.013 | 0.023 | 0.067 | -0.009 | 0.018 | 0.055 | -0.010 | 0.005 | 0.015 |
| t-tau | -0.017 | 0.002 | 0.005 | -0.012 | 0.003 | 0.008 | -0.010 | 0.010 | 0.029 | -0.017 | 0.003 | 0.010 | -0.010 | 0.009 | 0.027 | -0.011 | 0.003 | 0.010 |
| Ventricular | -0.001 | 0.599 | 1.000 | -0.001 | 0.719 | 1.000 | -0.001 | 0.568 | 1.000 | 0.000 | 0.876 | 1.000 | -0.002 | 0.349 | 1.000 | -0.001 | 0.752 | 1.000 |
| Hippocampus | 0.000 | 0.950 | 1.000 | 0.002 | 0.558 | 1.000 | 0.003 | 0.430 | 1.000 | 0.004 | 0.464 | 1.000 | 0.004 | 0.244 | 1.000 | 0.003 | 0.377 | 1.000 |
| Whole brain | 0.000 | 0.908 | 1.000 | 0.001 | 0.695 | 1.000 | 0.002 | 0.591 | 1.000 | 0.002 | 0.633 | 1.000 | 0.006 | 0.090 | 0.540 | 0.002 | 0.466 | 1.000 |
| Entorhinal | -0.005 | 0.382 | 1.000 | -0.005 | 0.253 | 1.000 | -0.002 | 0.698 | 1.000 | -0.003 | 0.711 | 1.000 | 0.001 | 0.751 | 1.000 | -0.003 | 0.477 | 1.000 |
| Fusiform | 0.008 | 0.149 | 0.892 | 0.003 | 0.474 | 1.000 | 0.004 | 0.278 | 1.000 | 0.004 | 0.483 | 1.000 | 0.007 | 0.106 | 0.638 | 0.000 | 0.909 | 1.000 |
| Middle temporal | 0.003 | 0.599 | 1.000 | 0.003 | 0.447 | 1.000 | 0.003 | 0.454 | 1.000 | 0.006 | 0.318 | 1.000 | 0.007 | 0.089 | 0.533 | 0.005 | 0.198 | 1.000 |

Age, gender, education and *APOE-ε4* carrier status adjusted multiple linear model. Adjusted p values are corrected for multiple comparisons (Bonferroni). ADAS-13 Alzheimer’ s disease Assessment Scale-13, ADNI, Alzheimer’s disease Neuroimaging Initiative, *APOE-ε4* apolipoprotein E4, Aβ42, amyloid β peptide 42, C1q, complement C1q subcomponent subunit B; C2, complement C2; C5, complement C5; C6, complement C6; C8B, complement component C8 beta chain; CFB, complement factor B; CDRSB, Clinical Dementia Rating Sum of Boxes; CSF, cerebrospinal fuid; EF, executive function; LAN, language; MEM, memory function; MMSE, Mini-Mental State Examination;p-tau phosphorylated tau, t-tau total tau.

## Table S8 Sensitivity analyses of CSF complement proteins with cognitive function, AD pathology and neuroimaging in mild cognitive impairment participants adjusted for full models.

| Variables | C1q | | | C2 | | | C5 | | | C6 | | | C8B | | | CFB | | |
| --- | --- | --- | --- | --- | --- | --- | --- | --- | --- | --- | --- | --- | --- | --- | --- | --- | --- | --- |
|  | β | p value | adjust-p value | β | p value | adjust-p value | β | p value | adjust-p value | β | p value | adjust-p value | β | p value | adjust-p value | β | p value | adjust-p value |
| MMSE | 0.014 | 0.205 | 1.000 | 0.023 | 0.002 | 0.012 | 0.020 | 0.008 | 0.046 | 0.026 | 0.036 | 0.217 | 0.017 | 0.055 | 0.328 | 0.018 | 0.015 | 0.088 |
| CDRSB | -0.034 | 0.002 | 0.013 | -0.017 | 0.021 | 0.123 | -0.019 | 0.010 | 0.061 | -0.023 | 0.057 | 0.344 | -0.011 | 0.213 | 1.000 | -0.018 | 0.018 | 0.107 |
| ADAS-13 | -0.033 | 0.001 | 0.008 | -0.031 | <0.001 | <0.001 | -0.025 | <0.001 | 0.002 | -0.034 | 0.003 | 0.018 | -0.021 | 0.011 | 0.064 | -0.022 | 0.002 | 0.009 |
| ADNI_MEM | 0.042 | <0.001 | 0.001 | 0.028 | <0.001 | 0.001 | 0.029 | <0.001 | 0.001 | 0.034 | 0.005 | 0.032 | 0.025 | 0.004 | 0.026 | 0.026 | 0.001 | 0.004 |
| ADNI_LAN | 0.024 | 0.057 | 0.340 | 0.026 | 0.002 | 0.011 | 0.017 | 0.047 | 0.282 | 0.024 | 0.097 | 0.579 | 0.016 | 0.116 | 0.696 | 0.016 | 0.070 | 0.421 |
| ADNI_EF | 0.030 | 0.011 | 0.064 | 0.025 | 0.002 | 0.013 | 0.024 | 0.002 | 0.014 | 0.026 | 0.055 | 0.329 | 0.020 | 0.032 | 0.192 | 0.018 | 0.026 | 0.156 |
| Aβ | 0.010 | 0.286 | 0.857 | 0.005 | 0.434 | 1.000 | 0.005 | 0.431 | 1.000 | 0.011 | 0.277 | 0.831 | 0.005 | 0.443 | 1.000 | 0.007 | 0.280 | 0.839 |
| p-tau | -0.006 | 0.326 | 0.978 | -0.005 | 0.249 | 0.746 | -0.007 | 0.084 | 0.252 | -0.009 | 0.172 | 0.516 | -0.007 | 0.096 | 0.289 | -0.006 | 0.141 | 0.422 |
| t-tau | -0.010 | 0.168 | 0.504 | -0.009 | 0.082 | 0.245 | -0.010 | 0.042 | 0.127 | -0.011 | 0.148 | 0.445 | -0.012 | 0.026 | 0.077 | -0.009 | 0.071 | 0.212 |
| Ventricular | -0.012 | 0.004 | 0.025 | -0.012 | <0.001 | <0.001 | -0.011 | <0.001 | 0.001 | -0.015 | 0.001 | 0.004 | -0.010 | 0.003 | 0.016 | -0.011 | <0.001 | <0.001 |
| Hippocampus | 0.013 | 0.083 | 0.496 | 0.012 | 0.017 | 0.099 | 0.007 | 0.174 | 1.000 | 0.010 | 0.212 | 1.000 | 0.008 | 0.142 | 0.850 | 0.010 | 0.037 | 0.224 |
| Whole brain | 0.015 | 0.029 | 0.176 | 0.013 | 0.004 | 0.025 | 0.009 | 0.047 | 0.281 | 0.012 | 0.115 | 0.687 | 0.009 | 0.076 | 0.455 | 0.009 | 0.054 | 0.325 |
| Entorhinal | 0.012 | 0.211 | 1.000 | 0.003 | 0.683 | 1.000 | 0.005 | 0.418 | 1.000 | 0.006 | 0.582 | 1.000 | 0.003 | 0.677 | 1.000 | 0.005 | 0.467 | 1.000 |
| Fusiform | 0.011 | 0.251 | 1.000 | 0.017 | 0.007 | 0.040 | 0.013 | 0.043 | 0.258 | 0.019 | 0.069 | 0.412 | 0.017 | 0.023 | 0.135 | 0.019 | 0.003 | 0.015 |
| Middle temporal | 0.008 | 0.570 | 1.000 | 0.026 | 0.002 | 0.013 | 0.021 | 0.019 | 0.112 | 0.025 | 0.085 | 0.508 | 0.023 | 0.028 | 0.169 | 0.025 | 0.003 | 0.020 |

Age, gender, education and *APOE-ε4* carrier status adjusted multiple linear model. Adjusted p values are corrected for multiple comparisons (Bonferroni). ADAS-13 Alzheimer’ s disease Assessment Scale-13, ADNI, Alzheimer’s disease Neuroimaging Initiative, *APOE-ε4* apolipoprotein E4, Aβ42, amyloid β peptide 42, C1q, complement C1q subcomponent subunit B; C2, complement C2; C5, complement C5; C6, complement C6; C8B, complement component C8 beta chain; CFB, complement factor B; CDRSB, Clinical Dementia Rating Sum of Boxes; CSF, cerebrospinal fuid; EF, executive function; LAN, language; MEM, memory function; MMSE, Mini-Mental State Examination;p-tau phosphorylated tau, t-tau total tau.

## Table S9 Mediating effects of regional brain structures on the association between CSF complement proteins and cognition.

| Complement proteins |  | **Direct effects** | | | | **Indirect effects** | | | | **Proportion of indirect effect** |
| --- | --- | --- | --- | --- | --- | --- | --- | --- | --- | --- |
|  |  | β | 95%CI | | p value | β | 95%CI | | p value | p value |
| CSF C1q |  |  |  |  |  |  |  |  |  |  |
|  | Ventricular |  |  |  |  |  |  |  |  |  |
|  | MMSE | -0.009 | -0.10 | 0.09 | 0.860 | 0.072 | 0.02 | 0.14 | 0.010 | 0.282 |
|  | CDRSB | -0.070 | -0.17 | 0.03 | 0.162 | -0.067 | -0.13 | -0.01 | 0.010 | 0.029 |
|  | ADAS | -0.014 | -0.10 | 0.08 | 0.761 | -0.070 | -0.13 | -0.02 | 0.012 | 0.135 |
|  | ADNI_MEM | 0.066 | -0.02 | 0.15 | 0.138 | 0.075 | 0.02 | 0.14 | 0.010 | 0.018 |
|  | ADNI_LAN | -0.055 | -0.15 | 0.04 | 0.258 | 0.068 | 0.01 | 0.13 | 0.012 | 0.969 |
|  | ADNI_EF | 0.010 | -0.08 | 0.10 | 0.831 | 0.075 | 0.02 | 0.14 | 0.012 | 0.169 |
| CSF C2 |  |  |  |  |  |  |  |  |  |  |
|  | Ventricular |  |  |  |  |  |  |  |  |  |
|  | MMSE | 0.010 | -0.06 | 0.08 | 0.780 | 0.082 | 0.04 | 0.13 | <0.001 | 0.013 |
|  | CDRSB | 0.014 | -0.05 | 0.08 | 0.690 | -0.085 | -0.13 | -0.04 | <0.001 | 0.063 |
|  | ADAS | -0.026 | -0.09 | 0.04 | 0.416 | -0.077 | -0.12 | -0.04 | <0.001 | 0.003 |
|  | ADNI_MEM | 0.014 | -0.05 | 0.07 | 0.647 | 0.090 | 0.05 | 0.14 | <0.001 | 0.002 |
|  | ADNI_LAN | 0.000 | -0.07 | 0.07 | 0.993 | 0.076 | 0.04 | 0.12 | <0.001 | 0.048 |
|  | ADNI_EF | 0.016 | -0.05 | 0.08 | 0.624 | 0.085 | 0.05 | 0.13 | <0.001 | 0.006 |
|  | Whole brain |  |  |  |  |  |  |  |  |  |
|  | MMSE | 0.073 | 0.01 | 0.14 | 0.027 | 0.019 | 0.00 | 0.05 | 0.032 | 0.061 |
|  | CDRSB | -0.051 | -0.12 | 0.02 | 0.144 | -0.021 | -0.05 | 0.00 | 0.030 | 0.091 |
|  | ADAS | -0.081 | -0.14 | -0.02 | 0.009 | -0.023 | -0.05 | 0.00 | 0.026 | 0.022 |
|  | ADNI_MEM | 0.080 | 0.02 | 0.14 | 0.012 | 0.023 | 0.00 | 0.05 | 0.025 | 0.017 |
|  | ADNI_LAN | 0.063 | 0.00 | 0.13 | 0.062 | 0.014 | 0.00 | 0.04 | 0.069 | 0.356 |
|  | ADNI_EF | 0.076 | 0.01 | 0.14 | 0.020 | 0.026 | 0.00 | 0.06 | 0.027 | 0.021 |
|  | Fusiform |  |  |  |  |  |  |  |  |  |
|  | MMSE | 0.101 | 0.03 | 0.17 | 0.006 | 0.042 | 0.01 | 0.09 | 0.028 | 0.018 |
|  | CDRSB | -0.080 | -0.15 | -0.01 | 0.021 | -0.038 | -0.08 | 0.00 | 0.029 | 0.022 |
|  | ADAS | -0.122 | -0.19 | -0.06 | 0.000 | -0.042 | -0.09 | -0.01 | 0.023 | 0.020 |
|  | ADNI_MEM | 0.116 | 0.05 | 0.18 | 0.000 | 0.044 | 0.01 | 0.09 | 0.026 | 0.019 |
|  | ADNI_LAN | 0.085 | 0.02 | 0.15 | 0.017 | 0.041 | 0.01 | 0.09 | 0.025 | 0.025 |
|  | ADNI_EF | 0.084 | 0.01 | 0.16 | 0.029 | 0.047 | 0.01 | 0.10 | 0.029 | 0.019 |
|  | Middle temporal |  |  |  |  |  |  |  |  |  |
|  | MMSE | 0.088 | 0.01 | 0.17 | 0.022 | 0.056 | 0.02 | 0.10 | <0.001 | 0.002 |
|  | CDRSB | -0.080 | -0.16 | 0.00 | 0.037 | -0.038 | -0.08 | -0.01 | 0.008 | 0.026 |
|  | ADAS | 0.108 | 0.04 | 0.18 | 0.002 | 0.052 | 0.02 | 0.10 | 0.000 | 0.001 |
|  | ADNI_MEM | 0.109 | 0.04 | 0.18 | 0.003 | 0.051 | 0.02 | 0.10 | <0.001 | 0.004 |
|  | ADNI_LAN | 0.075 | 0.00 | 0.15 | 0.055 | 0.052 | 0.02 | 0.10 | 0.001 | 0.012 |
|  | ADNI_EF | 0.061 | -0.02 | 0.14 | 0.120 | 0.071 | 0.03 | 0.12 | <0.001 | 0.006 |
| CSF C5 |  |  |  |  |  |  |  |  |  |  |
|  | Ventricular |  |  |  |  |  |  |  |  |  |
|  | MMSE | 0.009 | -0.05 | 0.07 | 0.788 | 0.069 | 0.03 | 0.11 | <0.001 | 0.029 |
|  | CDRSB | -0.004 | -0.07 | 0.06 | 0.915 | -0.068 | -0.11 | -0.03 | <0.001 | 0.037 |
|  | ADAS | -0.006 | -0.07 | 0.06 | 0.860 | -0.067 | -0.11 | -0.03 | <0.001 | 0.003 |
|  | ADNI_MEM | 0.022 | -0.04 | 0.08 | 0.455 | 0.074 | 0.04 | 0.12 | <0.001 | 0.036 |
|  | ADNI_LAN | -0.028 | -0.09 | 0.03 | 0.400 | 0.068 | 0.03 | 0.11 | <0.001 | 0.270 |
|  | ADNI_EF | 0.021 | -0.04 | 0.08 | 0.506 | 0.071 | 0.03 | 0.11 | <0.001 | 0.009 |
|  | Middle temporal |  |  |  |  |  |  |  |  |  |
|  | MMSE | 0.062 | -0.02 | 0.14 | 0.111 | 0.055 | 0.02 | 0.10 | 0.001 | 0.006 |
|  | CDRSB | -0.062 | -0.14 | 0.01 | 0.113 | -0.038 | -0.08 | -0.01 | 0.005 | 0.025 |
|  | ADAS | -0.069 | -0.14 | 0.00 | 0.066 | -0.054 | -0.10 | -0.02 | 0.001 | 0.002 |
|  | ADNI_MEM | 0.096 | 0.03 | 0.17 | 0.008 | 0.049 | 0.02 | 0.09 | 0.001 | 0.002 |
|  | ADNI_LAN | 0.016 | -0.06 | 0.09 | 0.675 | 0.055 | 0.02 | 0.10 | 0.002 | 0.107 |
|  | ADNI_EF | 0.046 | -0.03 | 0.12 | 0.262 | 0.067 | 0.02 | 0.12 | 0.001 | 0.010 |
| CSF CFB |  |  |  |  |  |  |  |  |  |  |
|  | Ventricular |  |  |  |  |  |  |  |  |  |
|  | MMSE | -0.004 | -0.07 | 0.06 | 0.888 | 0.062 | 0.03 | 0.10 | 0.001 | 0.102 |
|  | CDRSB | 0.010 | -0.05 | 0.07 | 0.770 | -0.061 | -0.10 | -0.03 | 0.001 | 0.160 |
|  | ADAS | 0.002 | -0.06 | 0.06 | 0.962 | -0.060 | -0.10 | -0.02 | <0.001 | 0.104 |
|  | ADNI_MEM | 0.005 | -0.05 | 0.06 | 0.867 | 0.067 | 0.03 | 0.11 | 0.001 | 0.037 |
|  | ADNI_LAN | -0.050 | -0.11 | 0.01 | 0.110 | 0.060 | 0.02 | 0.10 | <0.001 | 0.833 |
|  | ADNI_EF | -0.022 | -0.08 | 0.04 | 0.485 | 0.066 | 0.03 | 0.11 | 0.001 | 0.226 |
|  | Middle temporal |  |  |  |  |  |  |  |  |  |
|  | MMSE | 0.048 | -0.03 | 0.12 | 0.219 | 0.056 | 0.02 | 0.10 | 0.002 | 0.016 |
|  | CDRSB | -0.041 | -0.12 | 0.04 | 0.304 | -0.039 | -0.08 | -0.01 | 0.005 | 0.056 |
|  | ADAS | -0.056 | -0.13 | 0.02 | 0.135 | -0.054 | -0.10 | -0.02 | 0.003 | 0.005 |
|  | ADNI_MEM | 0.065 | -0.01 | 0.14 | 0.079 | 0.052 | 0.02 | 0.10 | 0.001 | 0.005 |
|  | ADNI_LAN | -0.022 | -0.10 | 0.06 | 0.572 | 0.058 | 0.02 | 0.11 | 0.002 | 0.446 |
|  | ADNI_EF | -0.006 | -0.08 | 0.07 | 0.876 | 0.071 | 0.02 | 0.13 | 0.002 | 0.150 |

Mediation analysis was adjusted for gender, age, education, and *APOE-ε4* status. ADAS-13, Alzheimer’s disease Assessment Scale-13; ADNI, Alzheimer’s disease Neuroimaging Initiative; C1q, complement C1q subcomponent subunit B; C2, complement C2; C5, complement C5; C6, complement C6; C8B, complement component C8 beta chain; CFB, complement factor B; CDRSB, Clinical Dementia Rating Sum of Boxes; EF, executive function; LAN, language; MCI, mild cognitive impairment; MEM, memory function; MMSE, Mini-Mental State Examination.

## Table S10 Association of CSF complement biomarkers with age, gender and *APOE*-ε4 presence.

|  | Age | | Gender (Male) | | *APOE-ε4* presence | |
| --- | --- | --- | --- | --- | --- | --- |
|  | β | p value | β | p value | β | p value |
| C1q | 0.052 | <0.0001 | 0.498 | <0.0001 | -0.056 | 0.580 |
| C2 | 0.025 | 0.017 | 0.473 | 0.001 | -0.140 | 0.194 |
| C5 | 0.022 | 0.032 | 0.452 | 0.001 | -0.067 | 0.536 |
| C6 | 0.021 | 0.042 | 0.282 | 0.047 | -0.144 | 0.191 |
| C8B | 0.017 | 0.104 | 0.379 | 0.008 | -0.059 | 0.595 |
| CFB | 0.007 | 0.494 | 0.392 | 0.006 | -0.212 | 0.053 |

Age, gender (female reference category), clinical diagnosis and *APOE-ε4* presence adjusted linear regression model. P-values are not adjusted for multiple comparisons.

## Table S11 Associations of CSF clusterin with longitudinal cognitive function, AD pathology and neuroimaging in cognitively normal and mild cognitive impairment participants.

| Variables | CN | | | MCI | | |
| --- | --- | --- | --- | --- | --- | --- |
|  | β | p value | adjust-pvalue | β | p value | adjust-p value |
| MMSE | -0.003 | 0.624 | 1.000 | 0.017 | 0.032 | 0.194 |
| CDRSB | 0.013 | 0.062 | 0.373 | -0.024 | 0.002 | 0.012 |
| ADAS-13 | -0.002 | 0.639 | 1.000 | -0.025 | <0.0001 | 0.003 |
| ADNI_MEM | -0.009 | 0.206 | 1.000 | 0.026 | 0.001 | 0.006 |
| ADNI_LAN | -0.002 | 0.820 | 1.000 | 0.016 | 0.067 | 0.401 |
| ADNI_EF | 0.004 | 0.621 | 1.000 | 0.025 | 0.002 | 0.014 |
| Aβ | -0.009 | 0.061 | 0.183 | 0.009 | 0.196 | 0.588 |
| p-tau | -0.004 | 0.279 | 0.837 | -0.009 | 0.035 | 0.106 |
| t-tau | -0.005 | 0.234 | 0.702 | -0.011 | 0.054 | 0.161 |
| Ventricular | 0.001 | 0.896 | 1.000 | -0.009 | 0.001 | 0.008 |
| Hippocampus | -0.002 | 0.669 | 1.000 | 0.009 | 0.075 | 0.449 |
| Whole brain | -0.001 | 0.859 | 1.000 | 0.014 | 0.003 | 0.016 |
| Entorhinal | -0.004 | 0.400 | 1.000 | 0.004 | 0.497 | 1.000 |
| Fusiform | 0.002 | 0.682 | 1.000 | 0.010 | 0.120 | 0.717 |
| Middle temporal | 0.003 | 0.524 | 1.000 | 0.019 | 0.037 | 0.221 |

Age, gender, education and *APOE-ε4* carrier status adjusted mixed effect models. Adjusted p values are corrected for multiple comparisons (Bonferroni). ADAS-13 Alzheimer’ s disease Assessment Scale-13, ADNI, Alzheimer’s disease Neuroimaging Initiative, *APOE-ε4* apolipoprotein E4, Aβ42, amyloid β peptide 42, CDRSB, Clinical Dementia Rating Sum of Boxes; CSF, cerebrospinal fuid; EF, executive function; LAN, language; MEM, memory function; MMSE, Mini-Mental State Examination;p-tau phosphorylated tau, t-tau total tau.

## Table S12 Mediating effects of regional brain structures on the association between CSF clusterin and cognition in mild cognitive impairment participants.

| Brain structure |  | **Direct effects** | | | | **Indirect effects** | | | | **Proportion of indirect effect** | |
| --- | --- | --- | --- | --- | --- | --- | --- | --- | --- | --- | --- |
|  |  | β | 95%CI | | p value | β | 95%CI | | p value | β | p value |
| Ventricular |  |  |  |  |  |  |  |  |  |  |  |
|  | MMSE | 0.058 | -0.003 | 0.120 | 0.063 | 0.053 | 0.020 | 0.090 | 0.001 | 0.475 | 0.002 |
|  | CDRSB | -0.050 | -0.110 | 0.010 | 0.107 | -0.053 | -0.092 | -0.020 | 0.001 | 0.514 | 0.003 |
|  | ADAS | -0.068 | -0.124 | -0.010 | 0.014 | -0.050 | -0.087 | -0.020 | <0.0001 | 0.423 | <0.0001 |
|  | ADNI_MEM | 0.051 | -0.005 | 0.110 | 0.070 | 0.060 | 0.025 | 0.100 | <0.0001 | 0.539 | <0.0001 |
|  | ADNI_LAN | 0.017 | -0.044 | 0.080 | 0.589 | 0.052 | 0.020 | 0.090 | 0.001 | 0.734 | 0.036 |
|  | ADNI_EF | 0.059 | 0.001 | 0.120 | 0.048 | 0.056 | 0.022 | 0.100 | <0.0001 | 0.482 | 0.001 |
| Whole brain |  |  |  |  |  |  |  |  |  |  |  |
|  | MMSE | 0.091 | 0.027 | 0.160 | 0.006 | 0.019 | 0.002 | 0.040 | 0.022 | 0.168 | 0.024 |
|  | CDRSB | -0.080 | -0.145 | -0.020 | 0.015 | -0.022 | -0.048 | 0.000 | 0.015 | 0.207 | 0.016 |
|  | ADAS | -0.095 | -0.156 | -0.030 | 0.001 | -0.024 | -0.050 | 0.000 | 0.008 | 0.193 | 0.008 |
|  | ADNI_MEM | 0.085 | 0.025 | 0.150 | 0.006 | 0.025 | 0.005 | 0.050 | 0.007 | 0.221 | 0.008 |
|  | ADNI_LAN | 0.053 | -0.012 | 0.120 | 0.114 | 0.015 | -0.001 | 0.040 | 0.068 | 0.204 | 0.097 |
|  | ADNI_EF | 0.088 | 0.026 | 0.150 | 0.006 | 0.028 | 0.006 | 0.060 | 0.010 | 0.235 | 0.010 |

Mediation analysis was adjusted for gender, age, education, and *APOE-ε4* status. ADAS-13, Alzheimer’s disease Assessment Scale-13; ADNI, Alzheimer’s disease Neuroimaging Initiative; CDRSB, Clinical Dementia Rating Sum of Boxes; EF, executive function; LAN, language; MCI, mild cognitive impairment; MEM, memory function; MMSE, Mini-Mental State Examination.
